# Neofunctionalization of “Juvenile Hormone Esterase Duplication” in Drosophila as an odorant-degrading enzyme towards food odorants

**DOI:** 10.1038/s41598-017-13015-w

**Published:** 2017-10-03

**Authors:** Claudia Steiner, Françoise Bozzolan, Nicolas Montagné, Martine Maïbèche, Thomas Chertemps

**Affiliations:** Sorbonne Universités, UPMC Univ Paris 06, UPEC, INRA, CNRS, IRD, Institute of Ecology and Environmental Sciences of Paris, Paris, France

## Abstract

Odorant degrading enzymes (ODEs) are thought to be responsible, at least in part, for olfactory signal termination in the chemosensory system by rapid degradation of odorants in the vicinity of the receptors. A carboxylesterase, specifically expressed in *Drosophila* antennae, called “juvenile hormone esterase duplication (JHEdup)” has been previously reported to hydrolyse different fruit esters *in vitro*. Here we functionally characterize JHEdup *in vivo*. We show that the *jhedup* gene is highly expressed in large basiconic sensilla that have been reported to detect several food esters. An electrophysiological analysis demonstrates that ab1A olfactory neurons of *jhedup* mutant flies exhibit an increased response to certain food acetates. Furthermore, mutant flies show a higher sensitivity towards the same odorants in behavioural assays. A phylogenetic analysis reveals that *jhedup* arose as a duplication of the juvenile hormone esterase gene during the evolution of Diptera, most likely in the ancestor of Schizophora, and has been conserved in all the 12 sequenced Drosophila species. *Jhedup* exhibits also an olfactory-predominant expression pattern in other Drosophila species. Our results support the implication of JHEdup in the degradation of food odorants in *D*. *melanogaster* and propose a neofunctionalization of this enzyme as a *bona fide* ODE in Drosophilids.

## Introduction

Olfaction is fundamental for the implementation of many insect behaviours like host plant and mating partner foraging, identification of suitable oviposition sites and predator avoidance. Due to the availability of new tools in functional genomics and transcriptomics great progress has been made in understanding molecular processes of odor detection at the periphery of the olfactory system (antennae and maxillary palps covered with olfactory sensilla)^1,2^ and information processing at the central nervous system^3–5^, which eventually triggers behaviours. The efficient detection of volatile molecules in olfactory sensilla requires the interplay of three steps: 1) the transport of the odor through the sensillum lymph mediated by odorant-binding proteins (OBPs), 2) the interaction of the odor with the corresponding olfactory receptors (ORs) which are expressed at the membrane of the olfactory receptor neurons (ORNs) and 3) the inactivation of the odor^6^. Odor ligand/OR^7–9^ interactions have been intensely studied and are therefore rather well established, contrary to odor ligand/OBP interactions^10–12^ and odor signal termination, which remain neither well investigated nor understood. How the odor signal is actually inactivated is still debated mainly between proponents of two hypotheses. One proposes that extracellular odorant-degrading enzymes (ODEs) are at least partly responsible for the rapid degradation of odors in the vicinity of the receptors, *i*.*e*. leading to odor metabolites which are no longer able to activate ORs^13,14^, whereas the other suggests that particular scavenger forms of OBPs or ORs to catalyze odor inactivation^15,16^. Such a scavenger role has been also proposed for a sensory neuron membrane protein (SNMP), present in pheromone-sensitive ORNs of *Drosophila melanogaster *and required for the detection of the sex pheromone cis-vaccenyl acetate (cVA)^17^.

The candidate ODEs identified to date belong to various detoxification enzyme families, including cytochrome P450s (CYPs), aldehyde oxidases (AOXs) and carboxylesterases (CCEs)^18–20^. An unexpected diversity has been revealed by recently characterized antennal transcriptomes of several lepidopteran, coleopteran and dipteran species, which contain a large number of detoxification enzymes belonging to these families, many of them constituting candidate ODEs. The first antennal ODE that has been identified and functionally characterized was the carboxylesterase ApolPDE in the silk moth *Antheraea polyphemus* ^13^. ApolPDE is secreted in the sensillar lymph and is able to degrade the female sex pheromone *in vitro* ^14,21^ (PDE – pheromone degrading enzyme). In the following years, several other antennal enzymes have been shown to metabolize not only sex pheromones (PDEs) but also plant volatiles (ODEs) *in vitro*. Among them, mainly lepidopteran and coleopteran CCEs^22–27^ but also a few lepidopteran AOXs^28–30^ and one coleopteran P450^31^. However, only a very few studies focused on their function *in vivo*, all of them in *Drosophila melanogaster*. The fatty acid desaturase Desat1 has been shown to play a role in sex pheromone discrimination of fruit flies^32^ while the P450 CYP6a20 seems involved in the regulation of aggressiveness^33^, but for both enzymes the actual substrates remain unknown, including their role in antennal functioning. In contrast the role of the CCE esterase 6 (Est6) has been described in more detail and has been shown to be involved in the physiological and behavioural responses to the *Drosophila *sex pheromone cVA and to ubiquitous acetates emitted by food sources^34,35^.

In addition to Est6, five other extracellular CCEs have been found in the antennal transcriptome of *D*. *melanogaster* and constitute ODEs^20^. Among them, the “juvenile hormone esterase duplication (*jhedup*) exhibited the highest expression level. *Jhedup* is a flanking gene duplication of the juvenile hormone esterase (JHE) gene with a 42% amino acid sequence similarity^36^. In contrast to JHE, which degrades juvenile hormone and plays therefore a crucial role in insect development^37^, little is known about the function of JHEdup. In 2007 Crone *et al*.^38^ showed that JHEdup is not able to hydrolyze juvenile hormone *in vitro* but rather artificial substrates like short chain esters of 4-methylumbelliferone. More recently, it has been demonstrated that recombinant JHEdup is also active towards several fruit esters, in particular short chain esters with side chains^20^, raising the question of its involvement in olfaction. In order to clarify the function of *jhedup* in *D*. *melanogaster*, we investigated its precise expression pattern in olfactory tissues using qPCR and fluorescent labelling, as well as its role in the detection of various food acetate esters applying electrophysiological and behavioural approaches on a mutant strain. Furthermore, we investigated the expression of *jhedup* in the olfactory organs of other *Drosophila* species to clarify whether *jhedup* could be associated with a neofunctionalization in olfaction in these species. We also analysed the phylogenetic relationship of *jhe* and *jhedup* within the Diptera, in order to determine when it arose for the first time during insect evolution.

## Results

### Jhedup expression in olfactory tissues

**Figure 1:**
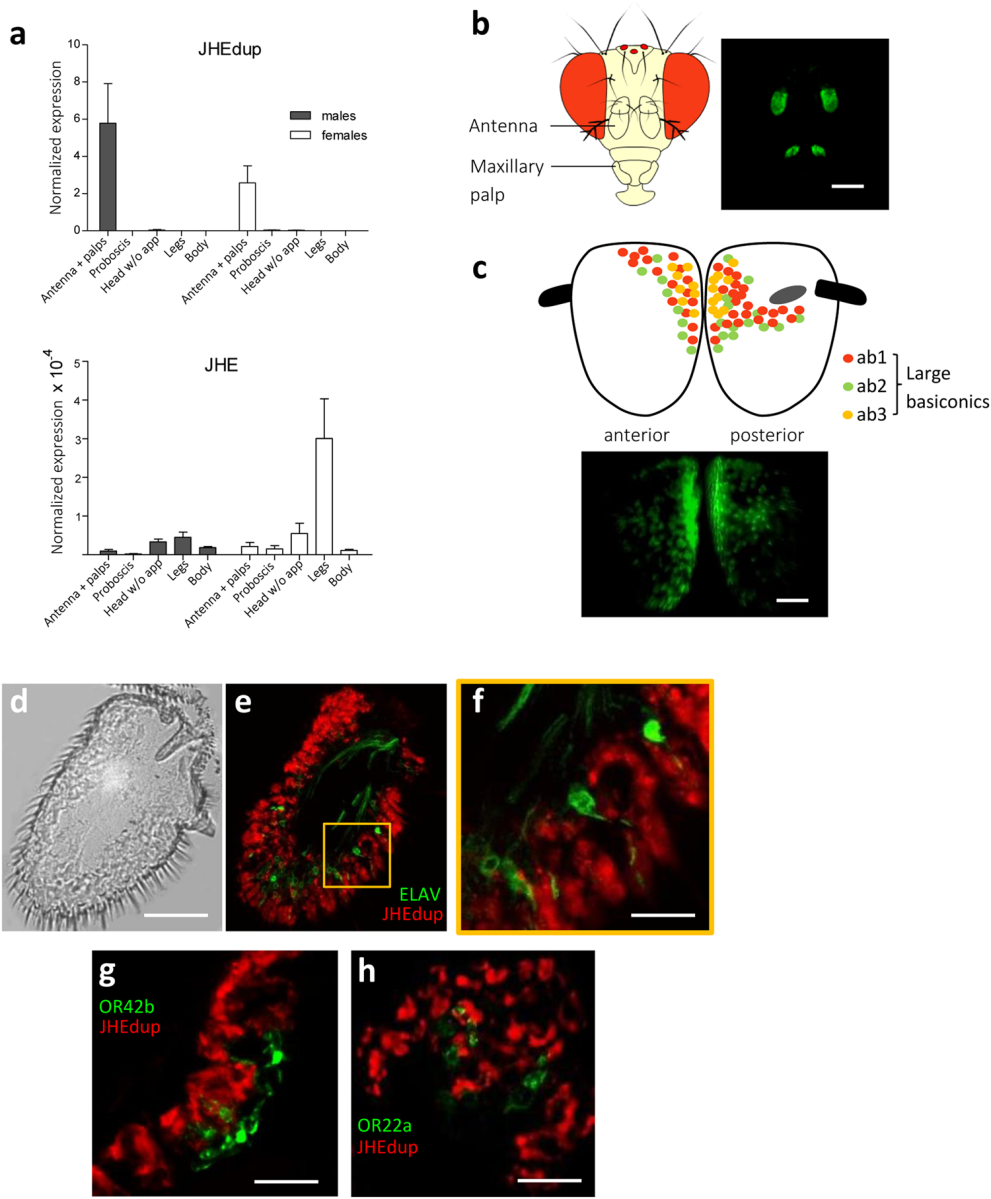
*Jhedup* expression in antennae. (**a**) Normalized expression of *jhedup* and *jhe* in antennae + maxillary palps, proboscises, heads without appendages, legs and bodies of wildtype (Canton-S) males and females using qPCR (reference gene for normalization of expression: *pgk*). The normalized expression level is indicated as mean ± SE of triplicate biological samples. (**b**) Expression of *jhedup* in antennae and maxillary palps of *jhedup-GAL4*; *UAS-mCD8::GFP* flies. Scale bar, 150 µm. (**c**) *jhedup* expression in the 3^rd^ antennal segment of *jhedup-GAL4*; *UAS-mCD8::GFP* flies (anterior and posterior view), including scheme for dispersion of large basiconics on the 3^rd^ antennal segment, scale bar 20 µm. (**d**) Antennal slice of 3^rd^ antennal segment. Scale bar, 20 µm. (**e**) *Jhedup* expression in 3^rd^ antennal segment of *jhedup-GAL4*; *UAS-mCD8::Cherry//elav-LexA*; *LexAOP-mCD8::GFP* flies. *Jhedup* expression (red), expression of neuronal marker *elav* (green). (**f**) Higher magnification of (**e**), Scale bar, 3 µm. *Jhedup* expression in 3^rd^ antennal segment of (**g**) *jhedup-GAL4*; *UAS-mCD8::Cherry//OR42b::GFP* and (**h**) *jhedup-GAL4*; *UAS-mCD8::Cherry//OR22a::GFP* flies. *Jhedup* expression (red), expression of olfactory receptor OR42b (ab1A neuron) or OR22a (ab3A neuron) coupled to GFP (green), detailed view. Scale bars (f/g), 6 µm.

Quantitative real-time PCR revealed a predominant expression of *jhedup* in the antennae and maxillary palps of both sexes which was 10.000 fold higher than the expression of the *jhe* gene (Fig. 1a). In contrast, *jhedup* expression was faint in the remaining body parts, including gustatory tissues such as proboscis and legs. No sexual dimorphism was observed. *In toto* observations of flies expressing green fluorescent protein (GFP) under the control of the *jhedup* promotor showed a strong labelling of the 3^rd^ antennal segments and maxillary palps, confirming an olfactory-restricted expression of *jhedup* (Fig. 1b). *Jhedup* is expressed broadly all over the 3^rd^ antennal segment but with a stronger expression in the area of the large basiconics (ab1, ab2 and ab3 sensilla) (Fig. 1c). Sections of *jhedup-GAL4*;*UAS-mCD8::Cherry//elav-LexA*; *LexAOP-mCD8::GFP* male and female antennae revealed that *jhedup* is expressed in nearly the whole antennal epithelium (Fig. 1d–f), in cells which do not express the neuronal marker ELAV. Additional labeling of ORs either expressed in ab1A (OR42b::GFP) or ab3A (OR22a::GFP) ORNs from basiconic sensilla revealed that *jhedup* is expressed in the close proximity of these ORNs (ab1A: Fig. 1g, ab 3A: Fig. 1h). Altogether, our results showed that *jhedup* is highly and broadly expressed in the third antennal segment, particularly in the area of the large basiconic sensilla (ab1, ab2 and ab3) and that it is at least associated with non-neuronal cells from ab1 and ab3 sensilla, either accessory cells surrounding the ORNs or epidermal cells.

### Impact of JHEdup on ORN response dynamics

**Figure 2:**
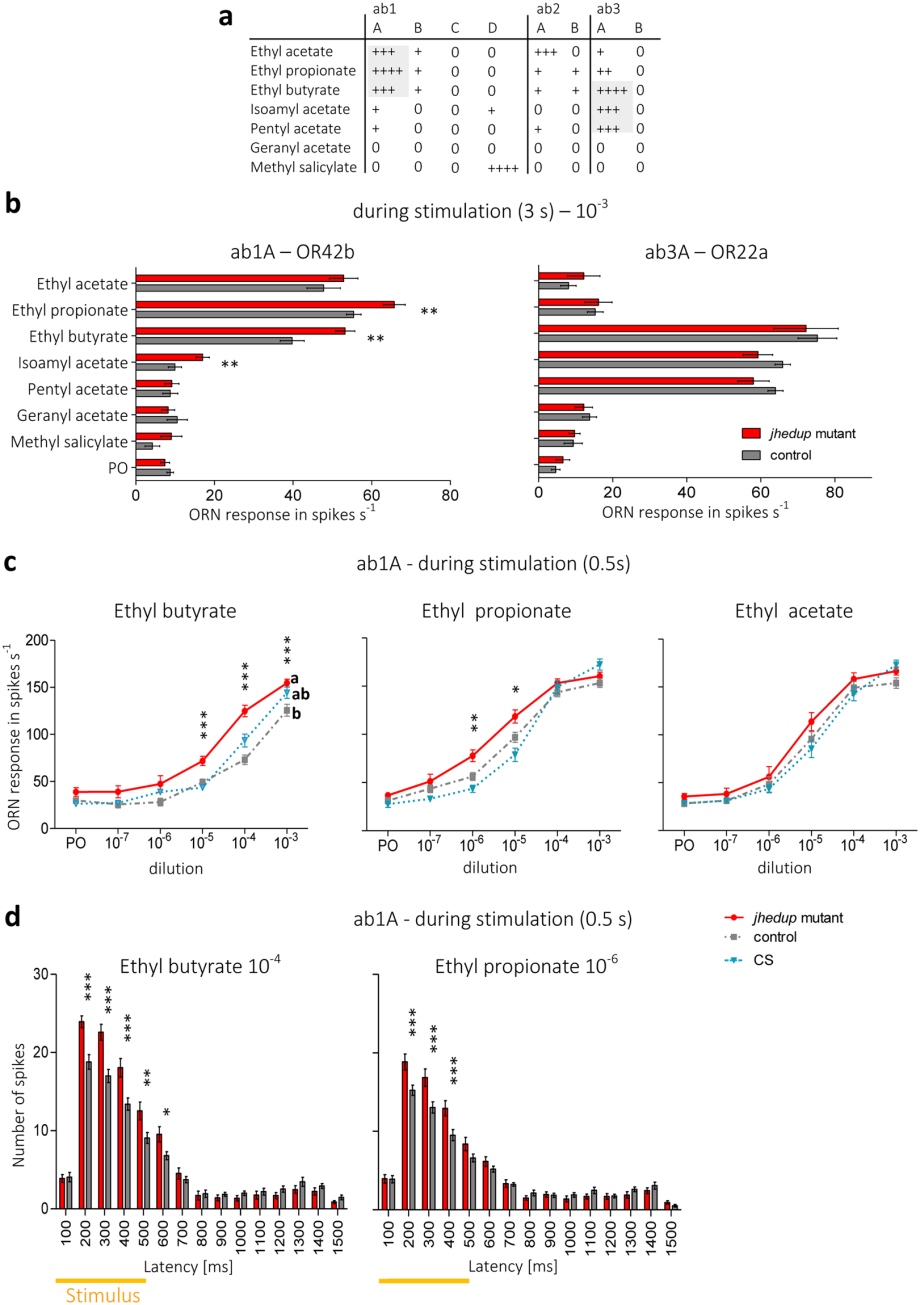
JHEdup’s involvement in physiological responses to food acetates. (**a**) Response pattern of large basiconic sensilla across a panel of seven acetates, modified from De Bruyne *et al*.^39^. (**b**) Single-sensillum responses of ab1 and ab3 sensilla of the *jhedup* mutant and the control strain to various food acetates; ORN response is shown in number of spikes per sec during the stimulation (3 s), mean ± SE (ab1: N ≥ 14 for each data point; ab3: N ≥ 9 for each data point), Mann Whitney U test. (**c**) Dose response curves of the three main ligands of OR42b (ethyl butyrate, ethyl propionate, ethyl acetate) of *jhedup* mutant, control and CS strains; ORN response is shown in number of spikes per second during the stimulation (0.5 s); mean ± SE (N ≥ 13 for each data point); Kruskal-Wallis test followed by Dunn’s multiple comparison post-hoc test (**d**) Peri-stimulus time histogramfor short stimulation of ethyl butyrate and ethyl propionate, concentrations of high differences between the genotypes were selected for this analysis. 2 way ANOVA, followed by a Bonferroni post-hoc test. PO: Paraffin oil, Data marked with different letters are significantly different; *P ≤ 0.05; **P ≤ 0.01; ***P ≤ 0.001.

ORNs from the large basiconic sensilla are known to detect common acetates released by fruits, especially ab1A and ab3A ORNs that detect a broad range of acetates^39^ (Fig. 2a). In order to test whether the responses to food acetates are modified in *jhedup* mutant flies (P ^*jhedup*^;Df^*jhedup*^) in comparison to control flies (CyO; Df^*jhedup*^ - same genetic background) we performed single-sensillum recordings (SSR) on ab1 and ab3 sensilla. Flies were first exposed to a panel of seven acetates for 3 s at high doses (10^–3^ dilution), this protocol of overstimulation was used in order to induce a high and saturated response of ORNs as done previously to show the role of Est6 in cVA detection^34^. In *jhedup* mutants the response intensity (number of action potentials per second) of ab3A neurons to the various acetates was not altered (Fig. 2b, right panel). In contrast, ab1A neurons of *jhedup* mutants showed an increased response intensity to ethyl butyrate, ethyl propionate and isoamyl acetate in comparison to the control strain, while the response to other food acetates remained unaffected (Fig. 2b, left panel).

To analyse in more detail the ab1A neuron responses, we performed a dose-response analysis for the three main ligands of the OR42b receptor, *i.e.* ethyl butyrate, ethyl propionate and ethyl acetate. At high doses and with long stimulation (3 s), ab1A neurons of *jhedup* mutants exhibited stronger responses to ethyl butyrate and ethyl propionate but not to ethyl acetate (Supplementary Fig. S1a), thus confirming our previous results (Fig. 2b). Thereafter, we shortened the stimulation time to 0.5s and performed likewise a dose-response analysis in order to verify whether ab1A neurons of *jhedup* mutants retain their increased responses to ethyl butyrate and ethyl propionate. Compared to the control strain, ab1A neurons of *jhedup *mutant flies showed stronger responses to ethyl butyrate for all the concentrations above the detection threshold (10^−5^ up to 10^−3^ dilution) (Fig. 2c). The same differences were observed when comparing with ab1A neurons of wild-type flies (Canton-S), except for the highest dose (10^−3^ dilution), a point that could be likely linked to their different genetical backgrounds. It has been demonstrated previously that diverse *Drosophila* wild-type strains are differently attracted to certain natural and synthetic compounds^40^. When stimulated with ethyl propionate, ab1A neurons of *jhedup* mutants also exhibited a higher spike frequency at low concentrations (10^−6^ and 10^−5^ dilution) (Fig. 2c), but these differences were abolished at higher doses (10^–4^ and 10^–3^ dilution) suggesting that high concentrations of ethyl propionate lead to a saturation level with equal maximum responses of ab1A neurons for all genotypes. Finally, ab1A responses to ethyl acetate were comparable at any dose in *jhedup* mutants and control strains, confirming the previous results (Fig. 2b and Supplementary Fig. S1a). *Jhedup* mutant ab1A neurons thus appear to exhibit a higher sensitivity to ethyl butyrate and ethyl propionate than the controls.

The response dynamics of ab1A neurons were then compared using a peri-stimulus time histogram (PSTH) analysis. The increased response intensity of the *jhedup *mutant flies to ethyl butyrate and ethyl propionate was only observed during the stimulation (0.5 s) (Fig. 2d) and the greatest differences were observed during the initial phase of the response, between 200 and 400 ms. For ethyl butyrate, differences were maintained until the end of the stimulation, up to 600 ms. For both odorants, the ab1A neuron firing rate returned to the basal level 200 ms after the stimulation (at about 700 ms) and this was similar between mutant and control flies. The PSTH analysis for long stimulations (3 s) showed that stronger responses of ab1A neurons of mutants to ethyl butyrate started instantly from 200–400 ms (similar to the observations during short stimulation), then declined markedly at around 900 ms and remained slightly above the average spontaneous ab1A firing rate. On the contrary, the increased response to ethyl propionate was delayed at 800 ms. Thus, slight differences occurred in the response dynamics to both odors (Supplementary Fig. S1b).

Overall, a depleted expression of *jhedup *in mutant flies led to an increased response of ab1A ORNs to some (ethyl butyrate, ethyl propionate) but not all (ethyl acetate) food acetates. In contrast, the response of mutant ab3A neurons to these acetates remained unaffected. The greater neuronal response in ab1 sensilla occurred mainly at the onset of the stimulation, whereas the response dynamics after the stimulation remained globally unaffected. Hence, JHEdup seems involved in the physiological dynamics of the response to certain acetates commonly released by fly food.

### Impact of JHEdup on behavioural response dynamics

**Figure 3:**
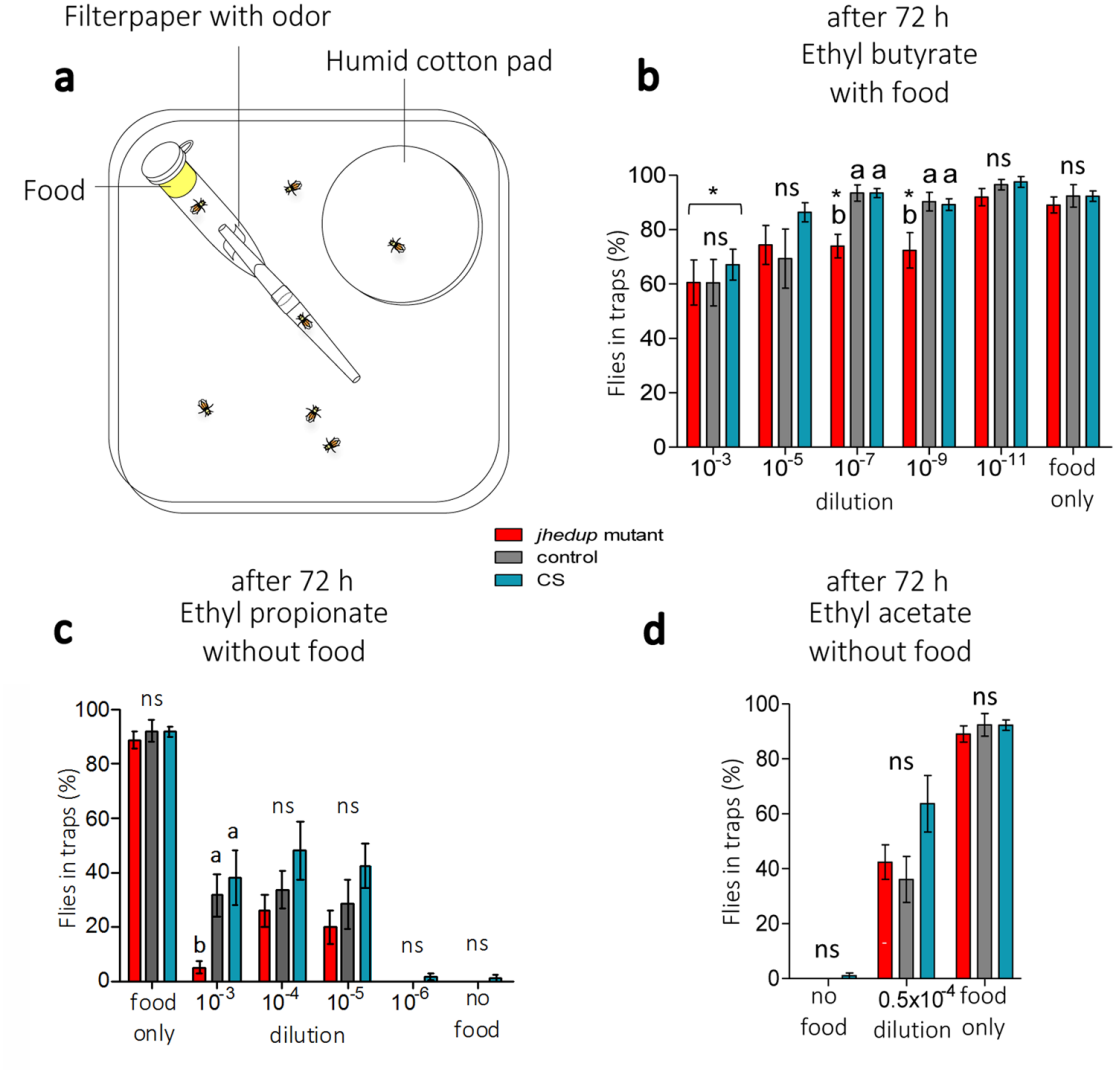
JHEdup’s function in behavioural response to certain food acetates. (**a**) Fly behaviour of *jhedup* mutant, control and CS was tested in a one trap assay. Food acetates were presented depending on their properties (repellent/attractant) in combination with or without regular food. Behavioural response is shown as number of flies in traps in percent, mean ± SE (N ≥ 10 traps for each data point ≈150 flies). Behavioural responses of non-starved females to (**b**) the repellent food acetate ethyl butyrate with food and the attractive food acetates (**c**) ethyl propionate and (**d**) ethyl acetate without food after 72 h. Kruskal-Wallis test, followed by a Dunn’s multiple comparison post-hoc test; data marked with different letters are significantly different, p < 0.05, ns: not significant. Asterisk indicates significantly different from food only.

As JHEdup seems involved in the ab1A ORN response to ethyl butyrate and ethyl propionate, we performed one trap assays (Fig. 3a) to investigate whether behavioural responses to these two food acetates could also be altered in adult *jhedup* mutants. First, we verified that empty traps were similarly avoided by mutant and control flies, demonstrating that flies did not enter the olfactory traps by hazard (Supplementary Fig. S2a). Flies of all genotypes detected and located food similarly (Supplementary Fig. S2b), indicating that olfactory-driven behaviours are not generally affected in *jhedup* mutant flies. Furthermore, the locomotor activity of mutant flies was similar to the controls (Supplementary Fig. S2c), showing that the *jhedup* mutation does not impair the fly’s locomotor abilities.

The individual odorant ethyl butyrate is known to trigger either aversion or attraction in *Drosophila* ^41^. These opposite behavioural responses might be dose-dependent^42,43^ as shown for other food odors^35,44^. In our trap assays, the attractiveness of regular fly food was diminished by 20% for all the genotypes in presence of ethyl butyrate at high concentration (10^−3^ dilution). This loss of food attractiveness was maintained also at lower concentrations (10^−7^, 10^−9^ dilution) for the *jhedup* mutant but not for the control strain and wildtype flies (Fig. 3b). Therefore, *jhedup* mutant flies seem more sensitive to ethyl butyrate than the control strains. Eventually, food attractiveness of the mutant flies could be restored at a very low concentration (10^−11^) and was comparable between all fly strains.

Attraction of insects, including Drosophila, to the fruit odors ethyl propionate^43,45,46^ and ethyl acetate^40,47^ has been demonstrated before, thus these acetates were presented in the trap without food. Low doses of ethyl propionate (10^−4^ dilution) and ethyl acetate (0.5 × 10^−4^ dilution) triggered attraction in all fly genotypes, with a maximum attraction of around 50% for ethyl propionate and 60% for ethyl acetate in wild-type flies (Fig. 3c,d). This is less than what we observed with fly food which leads to a maximum attraction of 90% under the same conditions. However, single odorants are known to be generally less attractive to insects than odor blends or natural food sources^48,49^. Interestingly, the attraction of *jhedup* mutant flies strongly decreased when increasing the dose of ethyl propionate (10^-3^ dilution) (less than 5%), whereas control and wild-type flies remained attracted (Fig. 3c). The reduced expression of *jhedup *in mutants seems to lead to a modified sensitivity of the flies to ethyl propionate, as observed for ethyl butyrate. On the other hand, all genotypes were equally attracted to ethyl acetate (Fig. 3d), which is consistent with the results obtained in SSR experiments. Ethyl acetate is known to be toxic for insects and was therefore only tested at a very low concentration^50–52^.

In conclusion, the greatly diminished expression of *jhedup* alters the sensitivity of mutant flies to the food acetates ethyl butyrate and ethyl propionate, neither affecting the olfactory system nor the locomotive activity in general.

### Evolutionary history of JHEdup

**Figure 4:**
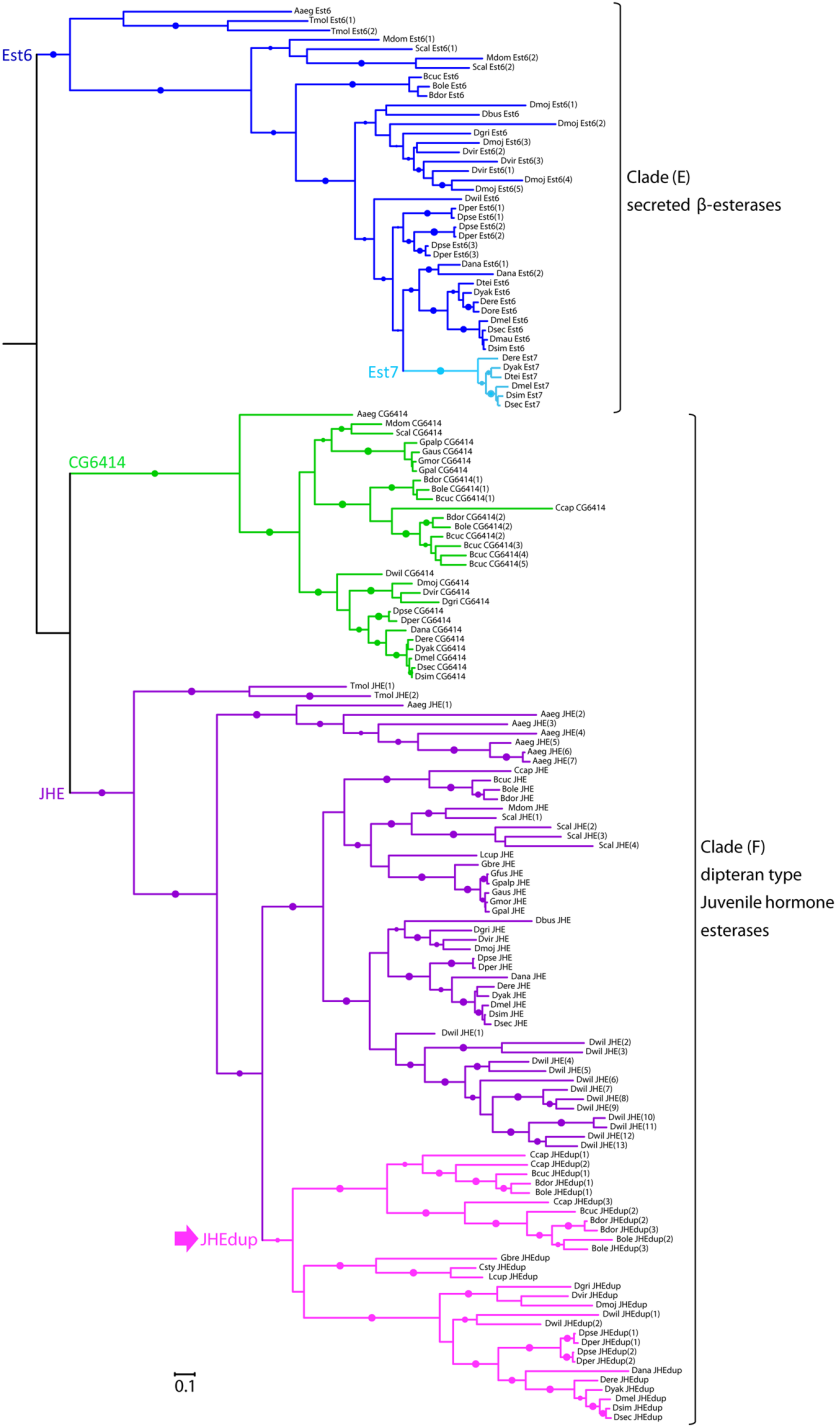
Evolutionary relationships of dipteran JHEdups, JHEs and other β-esterases. Phylogeny was created from full-length protein sequences applying the maximum likelihood method. The β-esterases esterase 6 and CG6414 were used as out groups. Dots represent branch support values based on the fast likelihood method, aLRT ≥ 0.9; aLRT < 0.9 were discarded. The branch length corresponds to the number of amino acid substitutions, *scale bar* indicates the average *number of amino acid substitutions* per residue.Aaeg, *Aedes aegypti*; Bcuc, *Bactrocera cucurbitae*; Bdor, *Bactrocera dorsalis*; Bole, *Bactrocera oleae*; Ccap, *Ceratitis capitata*; Csty, *Calliphora stygia*; Dana, *Drosophila ananassae*; Dbus, *Drosophila busckii*; Dere, *Drosophila erecta*; Dgri, *Drosophila grimshawi*; Dmau, *Drosophila mauritania*; Dmel, *Drosophila melanogaster*; Dmoj, *Drosophila mojavensis*; Dore, *Drosophila orena*; Dper, *Drosophila persimilis*; Dpse, *Drosophila pseudoobscura*; Dsec, *Drosophila sechellia*; Dsim, *Drosophila simulans*; Dtei, *Drosophila teissieri*; Dvir, *Drosophila virilis*; Dwil, *Drosophila willistoni*; Dyak, *Drosophila yakuba*; Gaus, *Glossina austeni*; Gbre, *Glossina brevipalpis*; Gmor, *Glossina morsitans*; Gpal, *Glossina pallidipes*; Gpalp, *Glossina palpalis*; Lcup, *Lucilia cuprina*; Mdom, *Musca domestica*; Scal, *Stomoxys calcitrans*; Tmol, *Tenebrio molitor*.

To investigate the evolutionary history of *jhedup* we carried out a phylogenetic analysis of dipteran β-esterases (considering the members of clades E and F in the insect esterase classification system^53^). We first searched for dipteran homologues of *D*. *melanogaster* *esterase* 6 (*est6*), *CG6414*, *jhe* and *jhedup* in available genomes and transcriptomes. Then, we built a maximum-likelihood phylogeny based on 182 amino acid sequences identified in Diptera. β-esterase sequences from the dipteran *Aedes aegypti* and the coleopteran *Tenebrio molitor*, in which a functional JHE has been identified^54,55^, were also included in the analysis (Fig. 4). JHEdup is like JHE and Est6 an extracellular catalytic active β-esterase containing a N-terminal signal peptide for secretion. JHEdup homologues clustered in a well-supported clade (aLRT: 0.93) located within the JHE lineage. Compared with the *est6* and *CG6414* clades, *jhe* and *jhedup* genes have higher duplication rates (notably with seven *jhe* copies in *Aedes aegypti* and 13 *jhe* copies in *Drosophila willistoni*) and higher evolutionary rates, as mirrored by the relatively long branch lengths in the JHE clade of the phylogeny.

**Figure 5:**
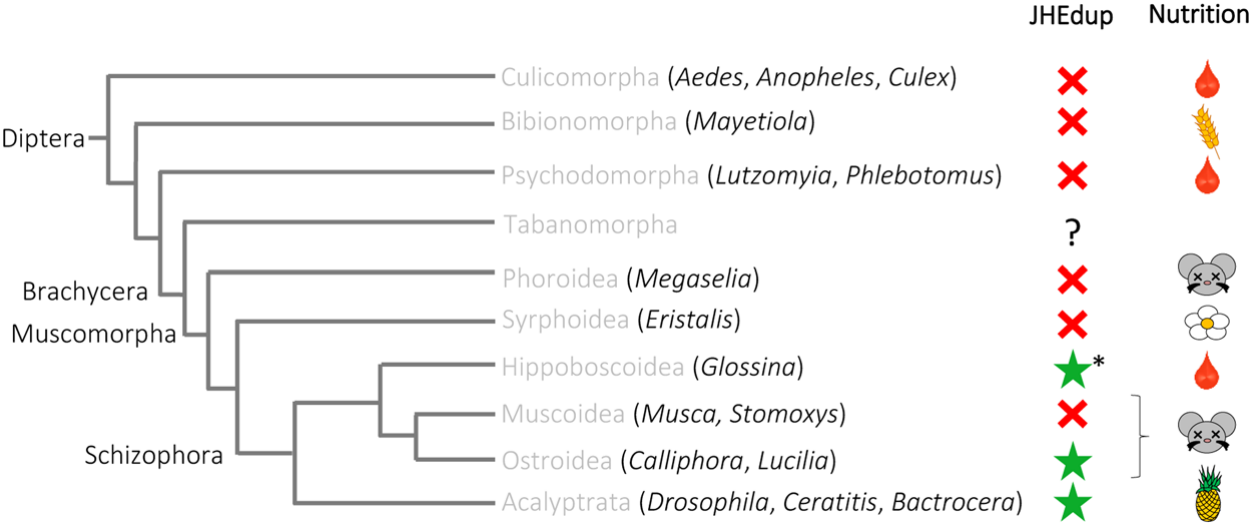
Distribution of JHEdup across the Diptera. Summary of phylogenetic relationships of Diptera adapted from Yeates and Wiegmann^85^. The red cross indicates absence of *jhedup*, the green star indicates the presence of *jhedup *in genome or transcriptome of various dipteran species, analyzed genera are indicated in brackets. Genomes and transcriptomes used for analysis come from NCBI database and VectorBase. *Jhedup* was not present in Culicomorpha, Bibionomorpha, Psychodomorpha, Phoroidea and Syrphoidea. *Jhedup* first evolved from a duplication of the JHE gene in the Brachycera lineage, before the emergence of the Schizophora. *Jhedup* was found in blood feeders (*G*. *brevipalpis*), carrion feeders (*C*. *stygia*, *L*. *cuprina*) and flies feeding on fruits (Drosophila, Ceratitis, Bactrocera). *Jhedup was found in one out of six Glossina species, in *G*. *brevipalpis*.

*Jhedup* genes were found not only in the 12 sequenced Drosophila species as shown previously^36^ but also in representatives of other fruit flies (*Ceratitis capitata*, *Bactrocera sp*.), blow flies (*Calliphora stygia*, *Lucilia cuprina*) and the tik-tik fly *Glossina brevipalpis*, all belonging to Schizophora (Figs 4 and 5). On the other hand, we found no *jhedup* homologue in Culicomorpha, Bibionomorpha, Psychodomorpha, Phoridae and Syrphidae (Fig. 5). Hence, we can conclude that *jhedup* arose from a duplication of the *jhe* gene that occurred likely in the common ancestor of Schizophora. Interestingly, *jhedup* was found in blood feeders (*G*. *brevipalpis*), carrion feeders (*C*. *stygia*, *L*. *cuprina*) and flies feeding on fruits (*Drosophila*, *Ceratitis*, *Bactrocera*), suggesting that JHEdup may not be linked to a particular lifestyle or feeding habit of these insects. However, *jhedup* was found in the genome of a single *Glossina* species amongst the six investigated and the JHEdup amino acid sequence of *G*. *brevipalpis* was lacking the signal peptide that is mandatory for secretion and might therefore be non-functional. Moreover, the presence of two or three *jhedup* genes in the fruit flies *Ceratitis capitata* and *Bactrocera sp*. indicates that several duplications of *jhedup* occurred in these fruit flies (Fig. 4).

### Jhedup expression in various Drosophila species

**Figure 6:**
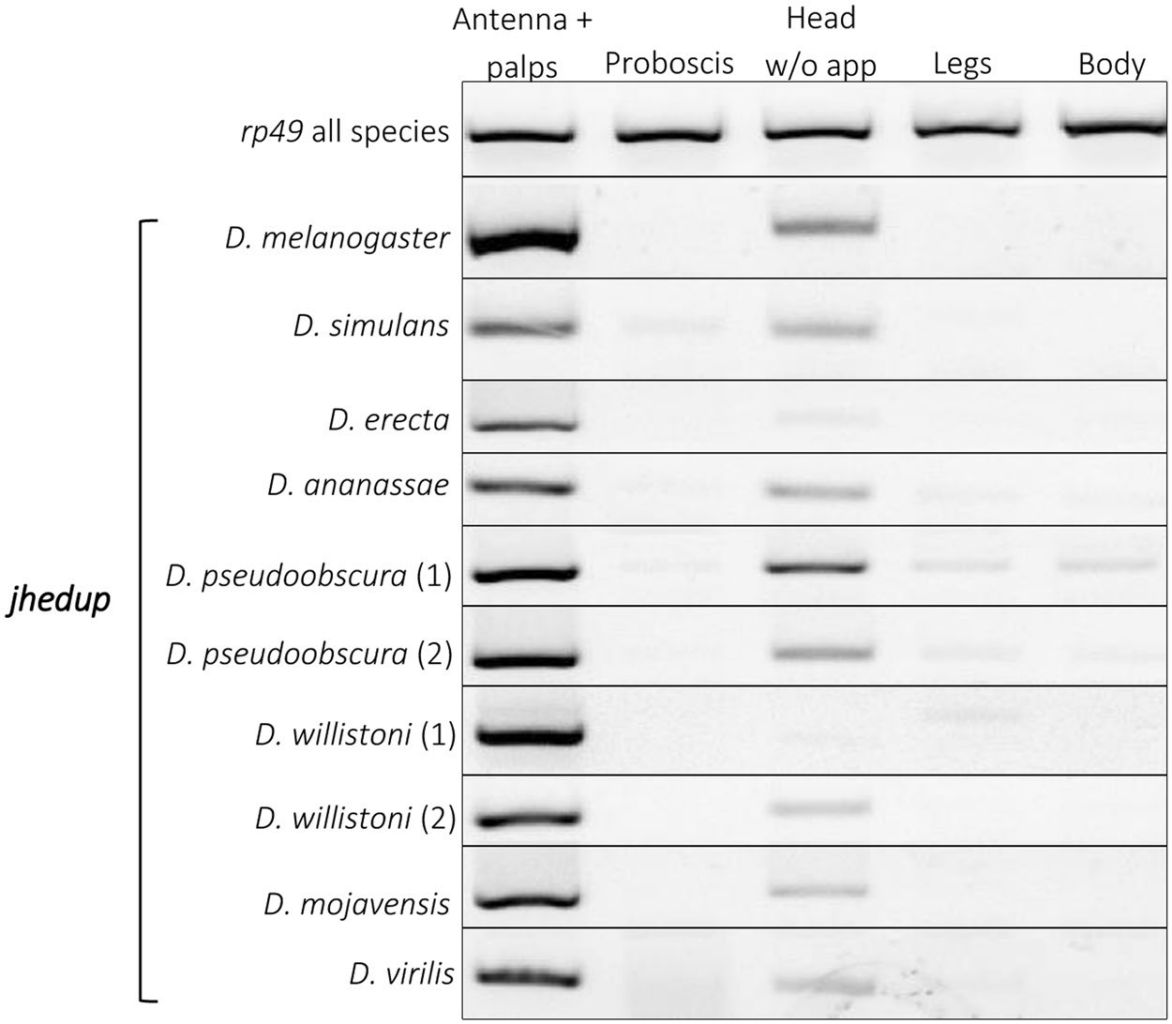
*Jhedup* expression among various Drosophila species. Expression pattern of *jhedup* in antennae, proboscises, heads without appendages, legs and bodies from adults of several Drosophila species (males and females mixed) using RT-PCR. The reference gene *rp49* was used to ensure quality of the cDNAs. Each delimited gel represents a representative view of *jhedup* expression in the given species. All drosophilid species have only one *jhedup* in their genome except *D*. *willistoni* and *D*. *pseudoobscura* with two isoforms.

In order to analyse a potential functional conservation of JHEdup, we investigated *jhedup* expression pattern in selected species across the genus *Drosophila *using RT-PCR. The predominant *jhedup* expression in antennae shown for *D*. *melanogaster *is highly consistent among all *Drosophila* species investigated (Fig. 6). Though, *jhedup* is also expressed in heads but much less intense. On the other hand, *jhedup* expression was absent or very faint in the remaining body parts including gustatory tissues like proboscis or legs.

### Comparison of JHEdup and JHE protein structure

**Figure 7:**
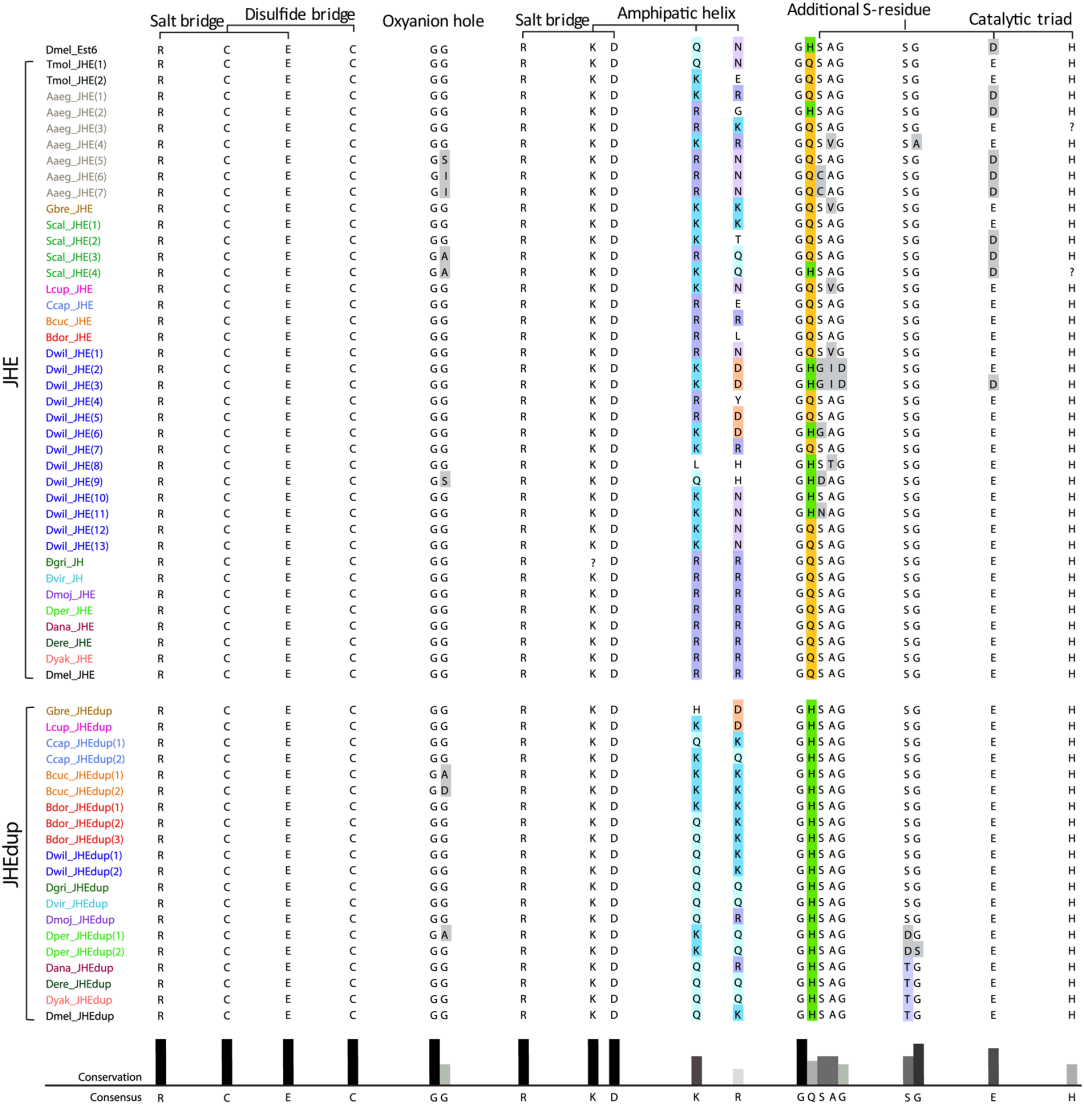
Comparison of domains and functional sites of JHEdup and JHE proteins. The domains and functional sites ofJHEdup and JHE are highly conserved. JHEdup and JHE differ just in one amino acid in the for carboxylesterases typical G**X**SXG motif surrounding the serine which is part of the catalytic triad. JHEdup has a G**H**SAG motif while JHE has a G**Q**SAG motif. JHEdup and JHE sequences of one species have the same color code. Coloured amino acids indicate sequence variations. X represents various amino acids depending on the type of esterase; G: Glycin; S: Serine; Q: Glutamine; A: Alanin; H: Histidin. Species abbreviations see Fig. 4.

Even though JHE and JHEdup sequences of several dipteran species are globally less conserved than other β-esterases, a comparison of important protein regions including sequences for protein structure (salt bridges, disulfide bridges) and sequences relevant for protein function (catalytic triad, oxyanion hole)^56–59^ revealed that those regions are very well conserved (Fig. 7). The only notable difference in the sequences of JHE and JHEdup is one amino acid in the carboxylesterase typical G**X**SXG motif surrounding the serine that is part of the catalytic triad. JHE has generally a G**Q**SAG motif while JHEdup has a G**H**SAG motif. Though, the GHSAG motif is not specific for JHEdup as it also occurs some JHEs of dipterans possessing several JHE duplications (*A*. *aegypti*, *Stomoxys calcitrans*, *D*. *willistoni*) and in other carboxylesterases like Est6. We further analyzed the protein structure of JHEdup and JHE of *D*. *melanogaster *using the prediction tool Phyre2. The primary protein structure of JHEdup consists of 559 amino acids, while JHE is a slightly larger protein comprising of 579 amino acids. Though, even if JHE and JHEdup exhibitonly 42% of amino acid sequence identity, the predicted secondary protein structure (alpha and beta-helices) is highly similar, including the positions of the oxyanion hole, the additional S-residue and the size of the binding pocket. Differences have been observed in the position ofglutamic acid (E), a member of the catalytic triad, which could potentially change the catalytic properties of the esterase including the suitability of certain substrates (Supplementary Fig. S4).

## Discussion

Here we characterized the CCE JHEdup which is involved in physiological and behavioural olfactory responses of Drosophila. *Jhedup* is specifically expressed in the olfactory organs (the third antennal segment and the maxillary palps) of Drosophila, thus supporting a specific role of this enzyme in olfaction. Indeed, such a restricted expression pattern has been previously shown to be a suitable criterion for the identification of insect olfactory-specific genes like ORs^1,8^, OBPs^60,61^ or ODEs/PDEs^26,28,29,62^. We found that *jhedup* expression is likely associated with accessory cells that have been previously suggested to produce ODEs and secret them in the sensillar lymph^34^ like it has been already demonstrated for OBPs^12,63,64^. The absence of sexual dimorphism in *jhedup* expression supported a function in the processing of food odorants rather than sex pheromones, as suggested by previous results with recombinant JHEdup enzyme^20^. Moreover, we found that *jhedup* is broadly expressed in the third antennal segment, especially in the area of the large basiconics, responsible for the detection of various food acetates^39^ that could be potential substrates of this CCE. Additionally *jhedup* is expressed in the maxillary palps also known to detect several food acetates^65^.

By an electrophysiological approach, we revealed that a depleted *jhedup* expression alters the response of ab1A ORNs to three food acetates (isoamyl acetate, ethyl butyrate, ethyl propionate), all short chain esters with side chains. A detailed analysis of the response dynamics showed that the response to those acetates was increased solely during stimulation in mutant flies. Moreover, *Jhedup* mutants show greater responses especially at the onset of the response in a range of a few hundred milliseconds (200–400 ms). This stays in contrast to the observations made for Est6 null mutants (lacking the *est6* gene) responding to the pheromone cVA and several food odors, where the antennal response was rather prolonged than more intense (higher spike frequency in SSR or a higher maximum amplitude in Electroantennography)^34,35^. In contrast responses of ab3A ORNs to our panel of acetates were not modulated in *jhedup* mutants, including the responses to isoamyl acetate and pentyl acetate that have previously been shown to be good substrates for recombinant JHEdup *in vitro* ^20^. This suggests that either *jhedup* is not expressed in ab3 sensilla, despite the close proximity observed here between jhedup expressing cells and ab3A neurons, or that another enzyme abundant in ab3 sensilla metabolizes the same odorants and thus compensates JHEdup’s function. *In vitro* studies have shown that recombinant JHEdup and Est6 are able to efficiently hydrolyse short chain esters with side chains like pentyl acetate or butyl acetate^20^. *Jhedup* and *est6* are both abundant in the third antennal segment and their expression is not restricted to a certain sensilla type^20,35^. In case both CCEs are present in ab3 sensilla, Est6 (or another abundant enzyme) could compensate the lack of JHEdup and mask or reduce the efficiency of the *jhedup* mutation. The rapidity of enzymatic degradation in sensilla, which should be very fast to allow an insect to efficiently navigate in a quickly changing olfactory environment, has always been a point of contention in the controversial debate about olfactory signal termination in vertebrates and insects. That enzymes located in antennae are able to rapidly degrade pheromones has been demonstrated in 1986 by Vogt *et al*.^66^ and confirmed in 2005 by Ishida and Leal^14^ for ApolPDE, a CCE of the wild silkmoth *Antheraea polyphemus *that metabolizes moth pheromone in a millisecond range. This was followed by other *in vitro *studies, especially on antennal CCEs, confirming their activity towards pheromones, though with less specificity than ApolPDE since those enzymes were metabolizing more efficiently host plant odors (ODEs)^22,23,25,26^. Thanks to these *in vitro* studies on various CCEs and on other enzyme families like AOXs, CYPs and GSTs^29–31,62^, evidence is gaining that PDEs/ODEs are indeed at least partly responsible for odorant inactivation and/or clearance in the vicinity of ORs. According to our data and previous biochemical studies, JHEdup could be considered as an ODE for food acetates in Drosophila.

Besides the physiological effect on the detection ofethyl butyrate and ethyl propionate, a depleted *jhedup* expression also modulated the fly behavioural response to these food acetates. Mutant flies exhibited a lower detection threshold to the repulsive fruit odor ethyl butyrate and were still repelled even at very low concentrations. High concentrations of the attractive odor ethyl propionate trigger a modified behaviour only in *jhedup* mutant flies. A similar behaviour has been observed in *est6* null mutants which had a lower detection threshold for the fruit odor pentyl acetate and were attracted and repelled at lower concentrations likened to the control flies^35^. *Jhedup* mutant flies seem to have a modified sensitivity to both odors.

If JHEdup is at least in part responsible for the degradation of certain food acetates in the sensillar lymph, the notably decreased expression of the CCE in mutants should cause an accumulation of those odors in the sensillar lymph which in turn could lead to an OR overstimulation and explain the observed oversensitivity of those flies to these odors.

It is known that gene duplications followed by gene diversification are a potential source of genetic novelties since mutations of duplicated genes are not affecting the function of the original genes^67^. In order to clarifythe origin of JHEdup duplication, we performed a phylogenetic analysis and compared several dipteran β-esterases like JHEdup, JHE, CG6414 and Est6. Those extracellular esterases are predicted to be catalytic competent and except CG6414, all have been functionally characterized in insects. Est6 was suggested to be involved in sensillar cVA and food odor processing^35,68,69^. JHE is known to be essential for insect development by degrading juvenile hormone^54,55^, whereas JHEdup does not^36^. We found that *jhedup* arose from a duplication of the *jhe* gene in Diptera, precisely in the Brachycera lineage in the common ancestor of Schizophora. Though, *jhedup* does not occur in all Schizophora. The absence of *jhedup* in Muscoidea suggests a secondary loss. Moreover, we found *jhedup* only in one out of six *Glossina* species, it was lacking the signal peptide for secretion and is therefore likely non-functional. This could also indicate a secondary loss of *jhedup* in the remaining Glossina species. A nutrition linked *jhedup* expression seems unlikely since *jhedup* occurs in blood feeders, carrion feeders and in flies feeding on fruits.

Interestingly, JHEdup and JHE are less conserved than Est6 and CG6414 as indicated by higher duplication events and higher evolutionary rates. Nevertheless, protein regions important for protein folding and catalytic activity are very well conserved. We found only one notable difference we found in the surroundings of one member of the catalytic triad (Serine-S), where JHEdup has a G**H**SAG motif instead of a G**Q**SAG motif, which is common for JHEs. For recombinant Est6 it has been demonstrated that replacing the positively charged histidine residue (**H**) with an uncharged glutamine residue (**Q**) with side-directed *in vitro* mutagenesis is not changing notably the biochemical properties of the enzyme^70^. In contrast, the exchange of the his-residue with a negatively charged glutamic acid residue (**E**) impacts dramatically enzyme properties like the pH optimum, the efficiency of hydrolyzing substrates and the Gibbs energy of activation. This suggests that the GHSAG motif of JHEdup might not be sufficient to explain the different substrate affinities with JHE. Furthermore, the predicted JHE and JHEdup protein structures are highly similar. We could just point out one difference in the position of one glutamic acid (**E**) in the binding pocket, which is an important member of the catalytic triad. This positional change could lead to varying preferences for certain substrates of JHEdup. Indeed, it is known that discrete changes around the active sites could modify the substrate specificity of various enzymes as reviewed in Todd *et al*.^71^.

Furthermore, we demonstrated that the predominant antennal expression pattern of *jhedup* is consistent across different Drosophila species, suggesting an olfactory-linked conserved function in fruit flies. *Est6* and *est-7* (also known as *est-5* or *est-p*), which is the duplication of *est6*, likewise exhibit different expression patterns^72^. While *est6* is present in all life stages, *est7* is mainly expressed in late larvae and in the adult stage, indicating a different function. These gene duplications are great examples of potential neofunctionalization associated to genome evolution.

Here we provide a comprehensive *in vivo* study of the candidate ODE JHEdup implementing an electrophysiological and behavioural approach. Conclusions from physiological observations on behaviour should be drawn cautiously but our electrophysiological data are consistent with the results obtained in behaviour. We propose JHEdup as candidate ODE in fly sensilla required for odorant processing. Certainly further investigations are mandatory for a better understanding of the single key players of olfactory peri-receptor events. However, our results strongly suggest a neofunctionalization of JHEdup in olfaction. From a more applied perspective, such ODEs could be considered as potential new targets for olfactory disruption-based strategies for the control of pest insects.

## Methods

### Fly strains

All fly lines were maintained on standard yeast-cornmeal-agar medium at 25 °C in a 12 h light/12 h dark cycle with a relative humidity of 60%.

For *in toto* localization of JHEdup in chemosensory tissues we used a transgenic fly line expressing mCD8::GFP under the control of the *jhedup* promotor. For the generation of JHEdup-Gal4 lines, the *jhedup* promotor region (367 bp fragment) was cloned in the pChs-Gal4 vector (primers described in Supplementary Table S1). Transgenic flies were generated by P-mediated germline transformation by BestGene Inc. (Chino Hill, USA). To specify *jhedup* expression, a *jhedup-GAL4*;*UAS-mCD8::Cherry* transgenic fly strain was created and crossed either with a strain expressing GFP under the *elav* promotor (*elav-LexA*;*LexAOP-mCD8::GFP*) or with strains in which the GFP is fused to an olfactory receptor belonging to a certain sensillum type (ab1A: *jhedupGAL4*;*UAS-mCD8::Cherry//OR42b::GFP* and ab3A: *jhedupGAL4*;*UAS-mCD8::Cherry//OR22a::GFP*).

For electrophysiological and behavioural experiments, flies were mutant for *jhedup* on one allele (P-element insertion in *jhedup*, DGRC 141268) and deficient due to a deletion in the *jhedup* locus on the other allele (DGRC 150036). Those mutant^*jhedup*^ flies (P^*jhedup*^;Df^*jhedup*^– test flies) resulted from a cross of a *jhedup *mutant parental line and a *jhedup* deficient line (Df^*jhedup*^; CyO), both provided by the Kyoto stock center. This crossing strategy allowed us to obtain, in the same progeny, *jhedup* mutants and control flies possessing the same genetical background. An 80% reduced expression of JHEdup in the P^*jhedup*^; Df^*jhedup*^ flies was confirmed with qPCR (Supplementary Fig. S3). Control flies were backcrossed with the wildtype Canton-S (CS). CS served as an additional control, despite different genetical backgrounds.

### Jhedup expression in olfactory tissues - qPCR

*Jhedup* and *jhe* transcripts were quantified by qPCR in various chemosensory tissues. RNA from 3–7 days old male and female antennae with maxillary palps, proboscises, legs, heads without appendages and fly bodies without appendages were extracted using TRIzol® Reagent (Invitrogen, USA). Single-stranded cDNAs were synthesized from total RNAs (1 μg) using Superscript II reverse transcriptase (Invitrogen, USA). All reactions were performed as previously described^73^. Each reaction was run in triplicate with at least three independent biological replicates. The *pgk* (FBgn0250906) was used as reference gene as in Chertemps *et al*.^34^. Specific primers and qPCR conditions are indicated in Supplementary Tables S1 and S2. Normalized *jhedup* expression was calculated with Q-Gene software^74^.

### Jhedup expression in various Drosophila species

*Jhedup* transcripts in various body tissues of several Drosophila species (*D*. *ananassae*, *D*. *erecta*, *D*. *melanogaster*, *D*. *mojavensis*, *D*. *pseudoobscura*, *D*. *simulans*, *D*. *virilis*, *D*. *willistoni*) were qualitatively analyzed by RT-PCR. RNA from 3–7 days old males and females (antennae with maxillary palps, proboscises, heads without appendages, legs, fly bodies without appendages) were extracted and cDNAs (25 ng) were synthesized as above. PCRs were performed using Q5® High-Fidelity DNA Polymerase (NEBiolabs, UK). PCR products were loaded on 1% agarose gels and visualized using Gel RED (VWR, USA) fluorescence. The *rp49* (FBgn0002626) was used as reference gene. Specific primers and RT-PCR conditions are indicated in Supplementary Tables S1 and S2.

### JHEdup expression in 3^rd^ antennal segment – Fluorescence microscopy

Antennae of 3–8 days old transgenic males and females were dissected and fixed for 45 min at room temperature (25 °C) in 4% paraformaldehyde with 0.3% Triton X-100 (Sigma-Aldrich, USA). Antennae were washed three times in phosphate buffered saline for 5 min, transferred in optimal cutting temperature (O.C.T.) embedding matrix (CellPath, UK), cut with a cryostat (Leica Biosystems, Germany) at −22 °C and transferred to SuperFrost®Plus microscope slides (VWR, Leuven). Antennal slices (6 µm) were mounted with ProLong® Gold antifade reagent with DAPI (Thermo-Fischer, USA) and were analysed under the laser-scanning confocal microscope (Leica TCS SP5 AOBS, Leica Biosystems, Germany). Pictures were analyzed and edited with ImageJ software.

### Impact of JHEdup on ORN response dynamics - Electrophysiology

Single sensillum recordings (SSR) were performed as previously described^75^ on ab1 and ab3 sensilla of 3–8 days old female *jhedup* mutant flies, control flies and the wild type Canton-S.

Stimulus cartridges consisted of a Pasteur pipette (Assistent, Germany) containing a filter paper (1cm × 0.5 cm; Sigma-Aldrich, USA). 10 µl of the odorant dilutions were applied, then the cartridges were immediately capped with a 1 ml pipette tip (STARLAB, France). Odorant dilutions (ethyl acetate, ethyl butyrate, ethyl propionate, geranyl acetate, isoamyl acetate, methyl salicylate, pentyl acetate; Sigma Aldrich, USA) using paraffin oil (Roth, Germany) as standard solvent were prepared in 10-fold steps (10^−1^ − 10^−7^). Cartridges were not used more than three times. Each odor has been tested only once on each fly.

The antenna was supplied with a constant charcoal-filtered humidified air flow (1.5 L.min^−1^ flux) delivered by a glass tube (ø0.7 cm) at a distance of approximately 1.5 cm. The odor stimuli were presented in pulses of 0.5 s or 3 s by inserting the stimulus cartridges in a hole placed 15 cm from the end of the glass tube delivering the constant air flow. The electrical signal was amplified using an EX-1 amplifier (Dagan Corporation, USA), sampled at 10 kHz through a Digidata 1440A acquisition board (Molecular Devices, USA) and filtered (high-pass: 1 Hz; low-pass: 3 kHz), to be finally recorded and analyzed with pCLAMP™ 10 software (Molecular Devices, USA). The net responses of ab1A and ab3A neurons were calculated by subtracting the spontaneous neuronal firing rate (spikes s^−1^), which was recorded before odor stimulation, from the firing rate during the stimulation (0.5 s/3 s). Peri-Stimulus Time Histogram (PSTH) analysis has been performed by counting the number of spikes in 100 ms bins.

### Impact of JHEdup on behavioural response dynamics

One trap assays were adapted from Woodard *et al*.^76^ and performed in square plastic containers (12 cm × 12 cm × 1.5 cm, Gosselin™, USA) (Fig. 3a) within a climate chamber: T: 25 °C, light-dark-cycle: 12 h/12 h, H: 70%. Traps were constructed from a 1.5 ml microfuge tube (Axygen®, USA) and two 300 µl micropipette tips (Biohitproline®tip, Finnland). Both pipette tips were cut 1.3 cm from the tip to create a diameter of ~0.2 cm, whereas one of the pipette tips was additionally cut at the broader end (1 cm). The two larger ends of the pipette tips were connected tightly and introduced in the tube which was previously cut at its narrow end (0.4 cm). A filterpaper (Millipore, Germany) with 100 µl of the tested odorant was placed around the introduced pipette tip. Odorants have been diluted in 10-fold steps in Milli-Q® water with Triton-X 100 (0.025%). Depending on whether the tested odorant is eliciting attractive (ethyl propionate, ethyl acetate) or repulsive behaviour (ethyl butyrate), approximately 300 µl of standard cornmeal medium (fly food) has been introduced on the opposite site of the trap. Afterwards the tubes have been closed and fixed in one corner of the containers. Then 15–20 non-starved female flies (4–9 days old) were slightly anesthetized with CO_2_ and put into the container. The number of flies in and outside the trap was counted after 72 h. This time range corresponds to two days of starvation to give flies a motivation to seek a food source to eventually count the number of flies in traps after 24 h.

### Statistical analysis

Statistical analyses have been performed with GraphPad Prism®5 software. For SSR of the seven acetates a two tailed T-test or Mann-Whitney U test were used depending on the normal distribution of the data. For SSR of ethyl butyrate, ethyl propionate and ethyl acetate ANOVAs were performed likewise according to the data distribution (parametric: one-way ANOVA, post-test Bonferroni’s multiple comparison; non parametric: Kruskal-Wallis test, post-test Dunn’s). The PSTH have been analyzed with a two-way ANOVA including a Bonferroni post-test. For behavioural data analysis we used a Kruskal-Wallis test with a Dunn’s post-hoc test and a two-way ANOVA with Bonferroni’s post-hoc test. Locomotor activity of the flies was analyzed with a one-way ANOVA including a Bonferroni’s multiple comparison post-hoc test. For the analysis of the depleted expression of *jhedup* in mutants we used a t-test.

### Bioinformatics and phylogeny

An exhaustive search for β-esterases sequences was carried outin 37 dipteran species, by searching NCBI database, VectorBase and FlyBase. Altogether, 25 incomplete or non-annotated sequences were (re) annotated with the GeneWise software^77^ using amino acid sequences of orthologous esterases as query. For the phylogeny 183 β-esterase amino acid sequences from the coleopteran *Tenebrio molitor* and from 37 dipteran species (sequences in Supplementary Table S3; *Anopheles gambiae*, *Culex quinquefasciatus*, *Mayetiola destructor*, *Lutzomyia longipalpis*, *Phlebotomus papatasi*, *Megaselia abdita* and *Eristalis dimidiata* not indicated in tree) were aligned using the online software MAFFT version7 ^78^ using the progressive method: G-INS-1 ^79^. Phylogenetic reconstruction was performed by applying the maximum likelihood method. The substitution model WAG + I + G + F^80^ was suggested as the best fitting model for protein evolution by ProtTest2.4 server^81^. Tree was created by using ATGC PhyML 3.0 bioinformatic platform^82^. For tree topology improvement both SPR (Subtree-Pruning-Regrafting) and NNI (Nearest-Neighbor-Interchange) methods were applied. The gamma shape parameter and the proportion of invariable sites were calculated by ProtTest2.4 server. Substitution rate categories were set as 4. Node support was estimated using a hierarchical likelihood-ratio test^83^. Tree editing was performed with iTOL software version3 ^84^. The presence of signal peptides in the amino acid sequences was verified using the online software SignalP 4.1 server.

## Electronic supplementary material

Supplementary data
