# Supplementary material for: Neofunctionalization of “Juvenile Hormone Esterase Duplication” in Drosophila as an odorant-degrading enzyme towards food odorants
Source: Sci Rep. 2017 Oct 3;7:12629. doi: 10.1038/s41598-017-13015-w (PMC5626784; doi:10.1038/s41598-017-13015-w)
Supplement: Supplementary file 1 — Supplementary data [file 41598_2017_13015_MOESM1_ESM.pdf]

Neofunctionalization of “Juvenile Hormone Esterase Duplication” in Drosophila as an odorant-degrading enzyme towards food odorants.

Claudia Steiner, Françoise Bozzolan, Nicolas Montagné, Martine Maïbèche and Thomas Chertemps

Table S1: Primer sequences for *jhedup*, *jhe* and the reference genes (*rp49*, *pkg*) for all Drosophila species used for RT-PCRs, qPCRs and the creation of Gal4 lines.

Oligo name	Sequence	Bases	Tm [°C]	Dvir	Dana	Dsim	Dmoj	Dere	Dwil	Dpse	Dmel
Reference gene - qPCR											
Pgk dir	CGA-GAA-ACT-GGT-GGA-GAA-GG	20	60								x
Pgk rev	CGA-AGT-TGG-GGA-ACT-CAA-AG	20	60								x
Reference gene - RT-PCR											
Rp49 dir	AGA-AGC-GCA-CCA-AGC-ACT-TCA-T	22	49,7	x	x	x	x	x	x	x	x
Rp49 rev1	AGG-AAC-TTC-TTG-AAT-CC	17	48		x	x		x			x
Rp49 rev2	AGG-AAT-TTC-TTG-AAG-CC	17	48	x			x		x	x	
Jhe - qPCR											
Jhe dir	GCT-GAC-CGT-CCA-AGG-GTT-TAT-GA	23	60								x
Jhe rev	AAG-TCG-GGA-AAG-AGC-AGT-GGA-CT	23	60								x
Jhedup - qPCR											
Jhedup dir	GCT-GGT-GAA-GGT-TCT-CTA-TTC-A	22	60								x
Jhedup rev	CAA-TTT-CAA-CAT-GGT-ATC-GTT-G	22	60								x
Jhedup – RT-PCR											
Jhedup dir2	CTT-GTG-TTT-GCA-GTC-GTC-GCT-CAT-G	25	54.2						x		
Jhedup dir3	TAC-CCA-AGG-CAT-TGA-GTA-TG	20	58						x		
Jhedup rev2	CAA-ACA-AGT-TCC-TCC-AGC-TT	20	58						x		
Jhedup rev3	TTT-CAG-CCA-TGT-TAA-AAG-AAT-TAG	24	43,7						x		
Jhedup dir4	GGC-CCT-GTT-TAG-TGG-TGC-ATC-GGT-AG	26	57,6							x	
Jhedup dir6	GCC-CCT-GTT-TAG-TGG-TTC-ATC-GGT-GG	26	57,6							x	
Jhedup rev4	TGT-ACC-ACT-GTG-GAA-GAT-CCC-GG	23	53,7							x	
Jhedup rev6	ATG-TGC-CAC-CGG-TTT-TGG-C	19	60							x	
Jhedup dirA	CAG-AGT-TCA-ATG-AGA-ACT-TCC-TGG	24	50,6					x			
Jhedup dirB	CCG-AGT-TCA-ATG-AAA-ACT-TCC-TGG	24	50,6			x					x
Jhedup dirC	AGG-AGT-TCA-ACG-AGA-ACT-TCC-TGG	24	52,3		x						
Jhedup dirD	AGG-AGT-TCA-ATG-CGA-ACT-TTT-TGG	24	48,9	x							
Jhedup dirE	AGG-AGT-TCA-ATG-AGA-ACT-TCT-TGG	24	48,9				x				
Jhedup revA	AGT-GGA-TTC-TCT-TCG-AGA-TT	20	56			x		x			x
Jhedup revB	AAG-GGA-TTC-TTT-TCG-AGA-TC	20	56		x						
Jhedup revC	TAT-AGA-TCG-AGA-CGG-GCT-GCT-GAG-C	25	55,9	x			x				
Jhedup promotor - creation of Gal4 lines											
Jhedup dir	GAA-TTC-CCG-GTA-GTC-ACT-TAG-TTG-TT	26	58								x
Jhedup rev	GGA-TCC-TTT-GTT-GTG-ATA-TCC-ATT-AG	26	58								x

Table S2: Conditions for RT-PCRs and qPCRs.

RT-PCR				
Species	rp49		jhedup	
	Primers	T [°C]	Primers	T [°C]
D. melanogaster	dir + rev1	50	dir B + rev A	56
D. virilis	dir + rev2	50	dir D + rev C	65
D. ananassae	dir + rev1	53	dir C + rev B	60
D. simulans	dir + rev1	53	dir B + rev A	56
D. mojavensis	dir + rev2	50	dir E + rev C	60
D. erecta	dir + rev1	53	dir A + rev A	56
D. willistoni	dir + rev2	50	dir 2 + rev 2	65
			dir 3 + rev 3	60
D. pseudoobscura	dir + rev2	50	dir 4 + rev 4	60
			dir 6 + rev 6	

qPCR						
Species	pgk		jhedup		jhe	
	Primers	T [°C]	Primers	T [°C]	Primers	T [°C]
D. melanogaster	dir + rev	60	dir + rev	60	dir + rev	60

Table S3: Sequences used for phylogenetic analysis.

	Code	Species	Original name	Database	Data source	Manual sequence change
JHEdup	Dmel JHEdup	*D. melanogaster*	NP_611085.2	NCBI	Genome	no
	Dsim JHEdup	*D. simulans*	EDX07298.1	NCBI - Genbank	Genome	no
	Dsec JHEdup	*D. sechellia*	FBgn0176540	Flybase	Genome	no
	Dyak JHEdup	*D. yakuba*	XP_002092277.1	NCBI	Genome	no
	Dere JHEdup	*D. erecta*	FBgn0112759	Flybase	Genome	yes
	Dana JHEdup	*D. ananassae*	FBpp0114484	Flybase	Genome	no
	Dpse JHEdup(1)	*D. pseudoobscura*	FBpp0336541	Flybase	Genome	no
	Dpse JHEdup(2)	*D. pseudoobscura*	FBpp0278754	Flybase	Genome	no
	Dper JHEdup(1)	*D. persimilis*	FBgn0158172	Flybase	Genome	no
	Dper JHEdup(2)	*D. persimilis*	FBgn0158183	Flybase	Genome	no
	Dvir JHEdup	*D. virilis*	XP_002050673.1	NCBI	Genome	no
	Dmoj JHEdup	*D. mojavensis*	FBgn0141893	Flybase	Genome	no
	Dgri JHEdup	*D. grimshawi*	FBgn0129530	Flybase	Genome	yes
	Dwil JHEdup (1)	*D. willistoni*	FBgn0223848	Flybase	Genome	no
	Dwil JHEdup (2)	*D. willistoni*	XP_002063356.1	NCBI	Genome	no
	Ccap JHEdup(1)	*Ceratitis capitata*	XP_004537660.1	NCBI	Genome	no
	Ccap JHEdup(2)	*Ceratitis capitata*	XP_004537547.1	NCBI	Genome	no
	Ccap JHEdup (3)	*Ceratitis capitata*	XP_004537546.1	NCBI	Genome	no
	Bcuc JHEdup(1)	*Bactrocera cucurbitae*	XP_011177853.1	NCBI	Genome	no
	Bcuc JHEdup(2)	*Bactrocera cucurbitae*	XP_011177856.1	NCBI	Genome	no
	Bdor JHEdup(1)	*Bactocera dorsalis*	XP_011208803.1	NCBI	Genome	no
	Bdor JHEdup(2)	*Bactocera dorsalis*	XP_011208804.1	NCBI	Genome	no
	Bdor JHEdup(3)	*Bactocera dorsalis*	XP_011208805.1	NCBI	Genome	no
	Bole JHEdup(1)	*Bactrocera oleae*	XP_014103601.1	NCBI	Genome	no
	Bole JHEdup(2)	*Bactrocera oleae*	XP_014103561.1	NCBI	Genome	no
	Bole JHEdup(3)	*Bactrocera oleae*	XP_014103593.1	NCBI	Genome	no
	Csty JHEdup	*Calliphora stygia*	AID61351.1	NCBI	Transcriptome	no
	Lcup JHEdup	*Lucilia cuprina*	KNC21239.1	NCBI	Genome	yes
JHE	Dmel JHE	*D. melanogaster*	FBpp0086362	Flybase	Genome	no
	Dsim JHE	*D. simulans*	FBpp0209563	Flybase	Genome	no
	Dsec JHE	*D. sechellia*	FBgn0176541	Flybase	Genome	no
	Dere JHE	*D. erecta*	FBpp0139115	Flybase	Genome	no
	Dyak JHE	*D. yakuba*	XP_002092276.1	NCBI	Genome	no
	Dana JHE	*D. ananassae*	FBpp0114485	Flybase	Genome	no
	Dpse JHE	*D. pseudoobscura*	FBpp0329953	Flybase	Genome	no
	Dper JHE	*D. persimilis*	FBgn0158194	Flybase	Genome	no
	Dgri JHE	*D. grimshawi*	XP_001987705.1	NCBI	Genome	yes
	Dvir JHE	*D. virilis*	XP_002050674.1	NCBI	Genome	no
	Dmoj JHE	*D. mojavensis*	XP_002005299.1	NCBI	Genome	no
	Dwil JHE(1)	*D. willistoni*	FBgn0223849	Flybase	Genome	no
	Dwil JHE(2)	*D. willistoni*	EDW74341.2	NCBI	Genome	no
	Dwil JHE(3)	*D. willistoni*	KRF97977.1	NCBI	Genome	yes
	Dwil JHE(4)	*D. willistoni*	FBgn0224857	NCBI	Genome	no
	Dwil JHE(5)	*D. willistoni*	XP_002074915.1	NCBI	Genome	no
	Dwil JHE(6)	*D. willistoni*	KRF97976.1	NCBI	Genome	yes
	Dwil JHE(7)	*D. willistoni*	EDW74338.2	NCBI	Genome	yes
	Dwil JHE(8)	*D. willistoni*	EDW74339.2	NCBI	Genome	no
	Dwil JHE(9)	*D. willistoni*	XP_002063354.1	NCBI	Genome	no
	Dwil JHE(10)	*D. willistoni*	KRF98354.1	NCBI	Genome	no
	Dwil JHE(11)	*D. willistoni*	KRF98355.1	NCBI	Genome	yes
	Dwil JHE(12)	*D. willistoni*	EDW75955.2	NCBI	Genome	no
	Dwil JHE(13)	*D. willistoni*	EDW75956.2	NCBI	Genome	no
	Ccap JHE	*Ceratitis capitata*	XP_004537544.1	NCBI	Genome	no
	Bcuc JHE	*Bactrocera cucurbitae*	XP_011178072.1	NCBI	Genome	yes
	Bdor JHE	*Bactocera dorsalis*	XP_011208806.1	NCBI	Genome	no
	Bole JHE	*Bactrocera oleae*	XP_014103560.1	NCBI	Genome	no
	Gaus JHE	*Glossina austeni*	GAUT025925	vectorbase	Genome	no
	Gbre JHE	*Glossina brevipalpis*	GBRI026559	vectorbase	Genome	yes
	Gfus JHE	*Glossina fuscipes*	GFUI004279	vectorbase	Genome	no
	Gmor JHE	*Glossina morsitans*	GMOY007669	vectorbase	Genome	no
	Gpal JHE	*Glossina pallidipes*	GPAI027742	vectorbase	Genome	no
	Gpalp JHE	*Glossina palpalis*	GPPI035309	vectorbase	Genome	yes
	Scal JHE(1)	*Stomoxys calcitrans*	XP_013119518.1	NCBI	Genome	no
	Scal JHE(2)	*Stomoxys calcitrans*	XP_013118542.1	NCBI	Genome	no
	Scal JHE(3)	*Stomoxys calcitrans*	XP_013119530.1	NCBI	Genome	no
	Scal JHE(4)	*Stomoxys calcitrans*	XP_013118535.1	NCBI	Genome	no
	Mdom JHE	*Musca domestica*	XP_005181511.1	NCBI	Genome	no
	Lcup JHE	*Lucilia cuprina*	KNC21240.1	NCBI	Genome	no
	Edim_JHE	*Eristalis dimidiata*		NCBI	publication	no

	Mabd_JHE	*Megaselia abdita*		NCBI	publication	no
	Ppap JHE(1)	*Phlebotomus papatasi*	PPAI010509-PA	vectorbase	Genome	yes
	Ppap JHE(2)	*Phlebotomus papatasi*	PPAI005680-PA	vectorbase	Genome	no
	Ppap JHE(3)	*Phlebotomus papatasi*	PPAI006912-RA	vectorbase	Genome	yes
	Ppap JHE(4)	*Phlebotomus papatasi*	PPAI005116-PA	vectorbase	Genome	yes
	Ppap JHE(5)	*Phlebotomus papatasi*	PPAI009850-PA	vectorbase	Genome	yes
	Ppap JHE(6)	*Phlebotomus papatasi*	PPAI006971-PA	vectorbase	Genome	yes
	Ppap JHE(7)	*Phlebotomus papatasi*	PPAI006829-RA	vectorbase	Genome	yes
	Llon JHE(1)	*Lutzomyia longipalpis*	LLOJ000101-PA	vectorbase	Genome	yes
	Llon JHE(2)	*Lutzomyia longipalpis*	LLOJ008548-PA	vectorbase	Genome	no
	Mdes_JHE	*Mayetiola destructor*		NCBI	publication	no
	Aaeg JHE(1)	*Aedes aegypti*	EAT43357.2	NCBI	Genome	no
	Aaeg JHE(2)	*Aedes aegypti*	XP_001648886.2	NCBI	Genome	no
	Aaeg JHE(3)	*Aedes aegypti*	XP_001650476.1	NCBI	Genome	no
	Aaeg JHE(4)	*Aedes aegypti*	XP_001650478.1	NCBI	Genome	no
	Aaeg JHE(5)	*Aedes aegypti*	XP_001650477.2	NCBI	Genome	no
	Aaeg JHE(6)	*Aedes aegypti*	XP_001647621.2	NCBI	Genome	no
	Aaeg JHE(7)	*Aedes aegypti*	XP_001650479.1	NCBI	Genome	no
	Cqui_JHE(1)	*Culex quinquefasciatus*	CPIJ002073-PA	vectorbase	Genome	no
	Cqui_JHE(2)	*Culex quinquefasciatus*	CPIJ007140-PA	vectorbase	Genome	no
	Cqui_JHE(3)	*Culex quinquefasciatus*	CPIJ013175-PA	vectorbase	Genome	no
	Cqui_JHE(4)	*Culex quinquefasciatus*	CPIJ013026-PA	vectorbase	Genome	no
	Cqui_JHE(5)	*Culex quinquefasciatus*	CPIJ013029-PA	vectorbase	Genome	no
	Cqui_JHE(6)	*Culex quinquefasciatus*	CPIJ002074-PA	vectorbase	Genome	no
	Cqui_JHE(7)	*Culex quinquefasciatus*	CPIJ013027-PA	vectorbase	Genome	no
	Cqui_JHE(8)	*Culex quinquefasciatus*	CPIJ007141-PA	vectorbase	Genome	no
	Cqui_JHE(9)	*Culex quinquefasciatus*	CPIJ014154-PA	vectorbase	Genome	no
	Cqui_JHE(10)	*Culex quinquefasciatus*	CPIJ002075-PA	vectorbase	Genome	no
	Agam_JHE(1)	*Anopheles gambiae*	AGAP005834-PA	vectorbase	Genome	no
	Agam_JHE(2)	*Anopheles gambiae*	AGAP005837-PA	vectorbase	Genome	no
	Agam_JHE(3)	*Anopheles gambiae*	AGAP005836-PA	vectorbase	Genome	no
	Agam_JHE(4)	*Anopheles gambiae*	AGAP005833-PA	vectorbase	Genome	no
	Agam_JHE(5)	*Anopheles gambiae*	AGAP005835-PA	vectorbase	Genome	no
	Tmol JHE(1)	*Tenebrio molitor*	AKZ17668.1	NCBI	unpublished	no
	Tmol JHE(2)	*Tenebrio molitor*	AAL41023.1	NCBI	publication	no
CG6414	Dmel CG6414	*D. melanogaster*	FBpp0401489	Flybase	Genome	no
	Dsim CG6414	*D. simulans*	KMZ08107.1	NCBI	Genome	no
	Dsec CG6414	*D. sechellia*	FBgn0167643	Flybase	Genome	no
	Dere CG6414	*D. erecta*	FBpp0137111	Flybase	Genome	no
	Dyak CG6414	*D. yakuba*	XP_002100141.2	NCBI	Genome	no
	Dana CG6414	*D. ananassae*	XP_001967782.1	NCBI	Genome	no
	Dpse CG6414	*D. pseudoobscura*	FBpp0275447	Flybase	Genome	no
	Dper CG6414	*D. persimilis*	FBgn0152149	Flybase	Genome	no
	Dgri CG6414	*D. grimshawi*	FBgn0132277	Flybase	Genome	no
	Dvir CG6414	*D. virilis*	FBgn0203615	Flybase	Genome	no
	Dmoj CG6414	*D. mojavensis*	XP_002011173.1	NCBI	Genome	no
	Dwil CG6414	*D. willistoni*	XP_002061222.1	NCBI	Genome	no
	Ccap CG6414	*Ceratitis capitata*	XP_004535154.1	NCBI	Genome	no
	Bcuc CG6414(1)	*Bactrocera cucurbitae*	XP_011185059.1	NCBI	Genome	no
	Bcuc CG6414(2)	*Bactrocera cucurbitae*	XP_011185054.1	NCBI	Genome	no
	Bcuc CG6414(3)	*Bactrocera cucurbitae*	XP_011185056.1	NCBI	Genome	no
	Bcuc CG6414(4)	*Bactrocera cucurbitae*	XP_011185055.1	NCBI	Genome	no
	Bcuc CG6414(5)	*Bactrocera cucurbitae*	XP_011185058.1	NCBI	Genome	no
	Bdor CG6414(1)	*Bactocera dorsalis*	XP_011200446.1	NCBI	Genome	no
	Bdor CG6414(2)	*Bactocera dorsalis*	XP_011200445.1	NCBI	Genome	no
	Bole CG6414(1)	*Bactrocera oleae*	XP_014088398.1	NCBI	Genome	no
	Bole CG6414(2)	*Bactrocera oleae*	XP_014088399.1	NCBI	Genome	no
	Gaus CG6414	*Glossina austeni*	GAUT001620	vectorbase	Genome	no
	Gmor CG6414	*Glossina morsitans*	GMOY010064	vectorbase	Genome	no
	Gpal CG6414	*Glossina pallidipes*	GPAI021827	vectorbase	Genome	yes
	Gpalp CG6414	*Glossina palpalis*	GPPI025999	vectorbase	Genome	no
	Scal CG6414	*Stomoxys calcitrans*	XP_013101285.1	NCBI	Genome	no
	Mdom CG6414	*Musca domestica*	XP_005181018.2	NCBI	Genome	yes
	Llon CG6414(1)	*Lutzomyia longipalpis*	LLOJ006979-PA	vectorbase	Genome	yes
	Llon CG6414(2)	*Lutzomyia longipalpis*	LLOJ007373PA	vectorbase	Genome	yes
	Llon_CG6414(3)	*Lutzomyia longipalpis*	LLOJ006902-PA	vectorbase	Genome	yes
	Aaeg CG6414	*Aedes aegypti*	XP_001663023.1	NCBI	Genome	no
	Agam_CG6414	*Anopheles gambiae*	AGAP001101-PA	vectorbase	Genome	no
Est6	Dmel Est6	*D. melanogaster*	AGB94442.1	NCBI	Genome	no
	Dmau Est6	*D. mauritiana*	AAA03158.1	NCBI	Genome	no
	Dsim Est6	*D. simulans*	FBpp0212768	Flybase	Genome	no
	Dsec Est6	*D. sechellia*	FBpp0206811	Flybase	Genome	no
	Dere Est6	*D. erecta*	FBpp0134113	Flybase	Genome	no
	Dyak Est6	*D. yakuba*	FBgn0239133	Flybase	Genome	no

	Dore Est6	*D. orena*	AAU05625.1	NCBI	Genome	no
	Dtei Est6	*D. teissieri*	AAU05621.1	NCBI	Genome	no
	Dana Est6(1)	*D. ananassae*	XP_001956019.1	NCBI	Genome	no
	Dana Est6(2)	*D. ananassae*	ACR09423.1	NCBI	Genome	no
	Dpse Est6(1)	*D. pseudoobscura*	AAB70225.1	NCBI	Genome	no
	Dpse Est6(2)	*D. pseudoobscura*	FBpp0274977	Flybase	Genome	no
	Dpse Est6(3)	*D. pseudoobscura*	FBpp0274978	Flybase	Genome	no
	Dper Est6(1)	*D. persimilis*	FBpp0189270	Flybase	Genome	no
	Dper Est6(2)	*D. persimilis*	FBgn0021251	Flybase	Genome	yes
	Dper Est6(3)	*D. persimilis*	FBpp0189269	Flybase	Genome	no
	Dgri Est6	*D. grimshawi*	FBgn0123005	Flybase	Genome	no
	Dvir Est6(1)	*D. virilis*	FBpp0224490	Flybase	Genome	no
	Dvir Est6(2)	*D. virilis*	FBpp0224491	Flybase	Genome	no
	Dvir Est6(3)	*D. virilis*	FBpp0224492	Flybase	Genome	no
	Dmoj Est6(1)	*D. mojavensis*	XP_002000510.1	NCBI	Genome	no
	Dmoj Est6(2)	*D. mojavensis*	KRG01848.1	NCBI	Genome	no
	Dmoj Est6(3)	*D. mojavensis*	FBgn0145204	Flybase	Genome	no
	Dmoj Est6(4)	*D. mojavensis*	FBgn0145202	Flybase	Genome	no
	Dmoj Est6(5)	*D. mojavensis*	FBgn0145201	Flybase	Genome	no
	Dwil Est6	*D. willistoni*	FBgn0227433	Flybase	Genome	no
	Dbus Est6	*D. busckii*	ALC45784.1	NCBI	Genome	yes
	Bcuc Est6	*Bactrocera cucurbitae*	XP_011196064.1	NCBI	Genome	no
	Bdor Est6	*Bactocera dorsalis*	XP_011199235.1	NCBI	Genome	no
	Bole Est6	*Bactrocera oleae*	XP_014094741.1	NCBI	Genome	no
	Scal Est6(1)	*Stomoxys calcitrans*	XP_013116073.1	NCBI	Genome	no
	Scal Est6(2)	*Stomoxys calcitrans*	XP_013116074.1	NCBI	Genome	no
	Mdom Est6(1)	*Musca domestica*	XP_005183940.1	NCBI	Genome	no
	Mdom Est6(2)	*Musca domestica*	XP_005183939.2	NCBI	Genome	no
	Llon Est6	*Lutzomyia longipalpis*	LLOJ001981-PA	vectorbase	Genome	no
	Aaeg Est6	*Aedes aegypti*	XP_001656119.1	NCBI	Genome	no
	Agam_Est6(1)	*Anopheles gambiae*	AGAP005372-PA	vectorbase	Genome	no
	Agam_Est6(2)	*Anopheles gambiae*	AGAP005371-PA	vectorbase	Genome	no
	Tmol Est6(1)	*Tenebrio molitor*	AKZ17673.1	NCBI	unpublished	no
	Tmol Est6(2)	*Tenebrio molitor*	AKZ17665.1	NCBI	unpublished	no
Est7	Dmel Est7	*D. melanogaster*	AAF49945.1	NCBI	Genome	no
	Dyak Est7	*D. yakuba*	FBpp0266903	Flybase	Genome	no
	Dere Est7	*D. erecta*	FBpp0134114	Flybase	Genome	no
	Dsim Est7	*D. simulans*	FBpp0212769	Flybase	Genome	no
	Dsec Est7	*D. sechellia*	FBpp0206812	Flybase	Genome	no
	Dtei Est7	*D. teissieri*	AAU05622.1	NCBI	Genome	no

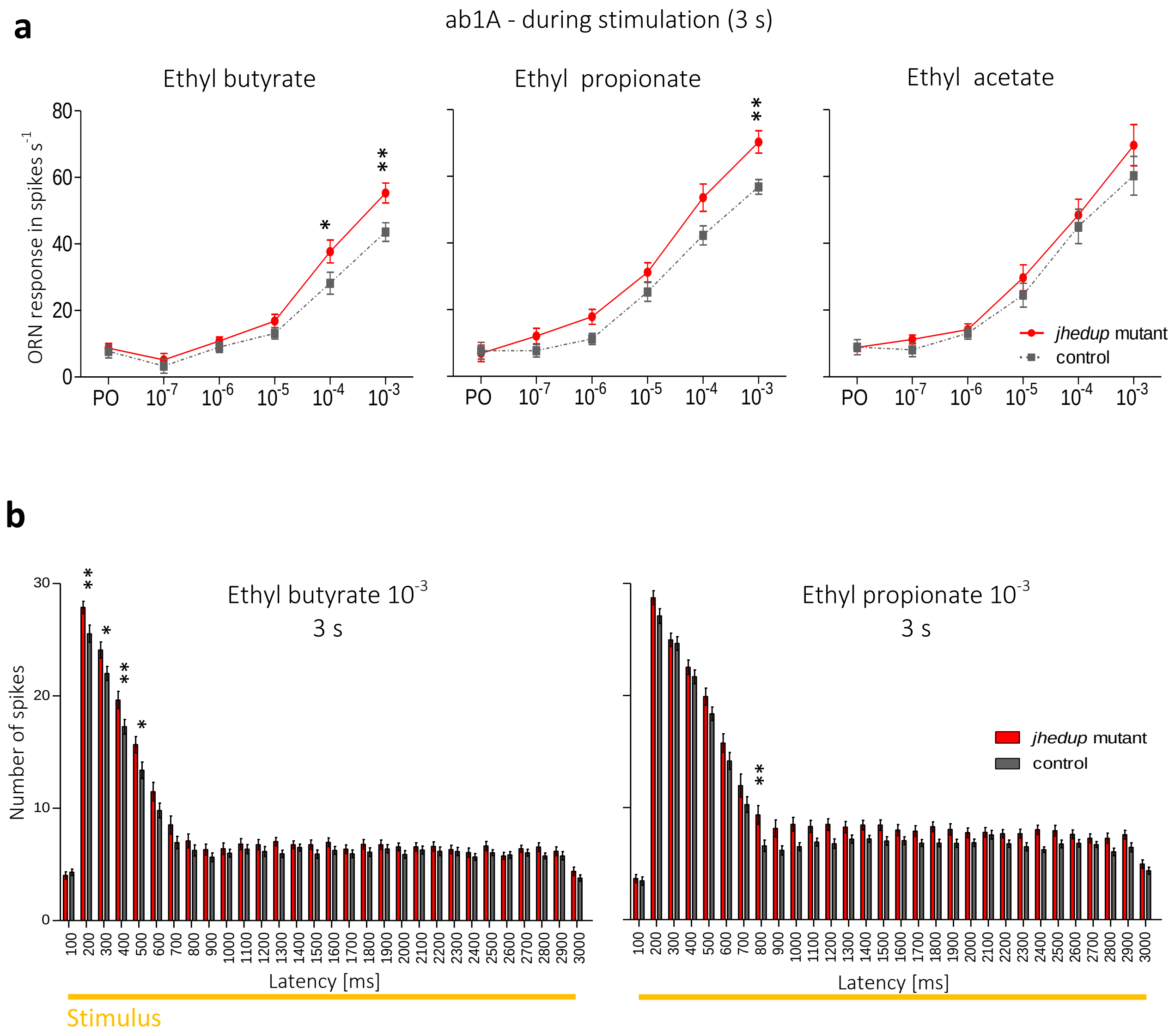

Figure S1. JHEdup's involvement in physiological response to food acetates. (a) Dose response curves of the three main ligands of OR42b (ethyl butyrate, ethyl propionate, ethyl acetate) of *jhedup* mutant and control, ORN response is shown in number of spikes per second during the stimulation (3 s); mean ± SE (N ≥ 13 for each data point); 1wayANOVA, post hoc: Bonferroni's multiple comparison; Kruskal-Wallis test, post hoc: Dunn's multiple comparison (b) peri-stimulus time histogram (PSTH) for 3 s stimulation of ethyl butyrate and ethyl propionate, selected for this analysis were concentrations of high differences between the genotypes. 2wayANOVA, post hoc: Bonferroni, *P ≤ 0.05; **P ≤ 0.01; ***P ≤ 0.001.

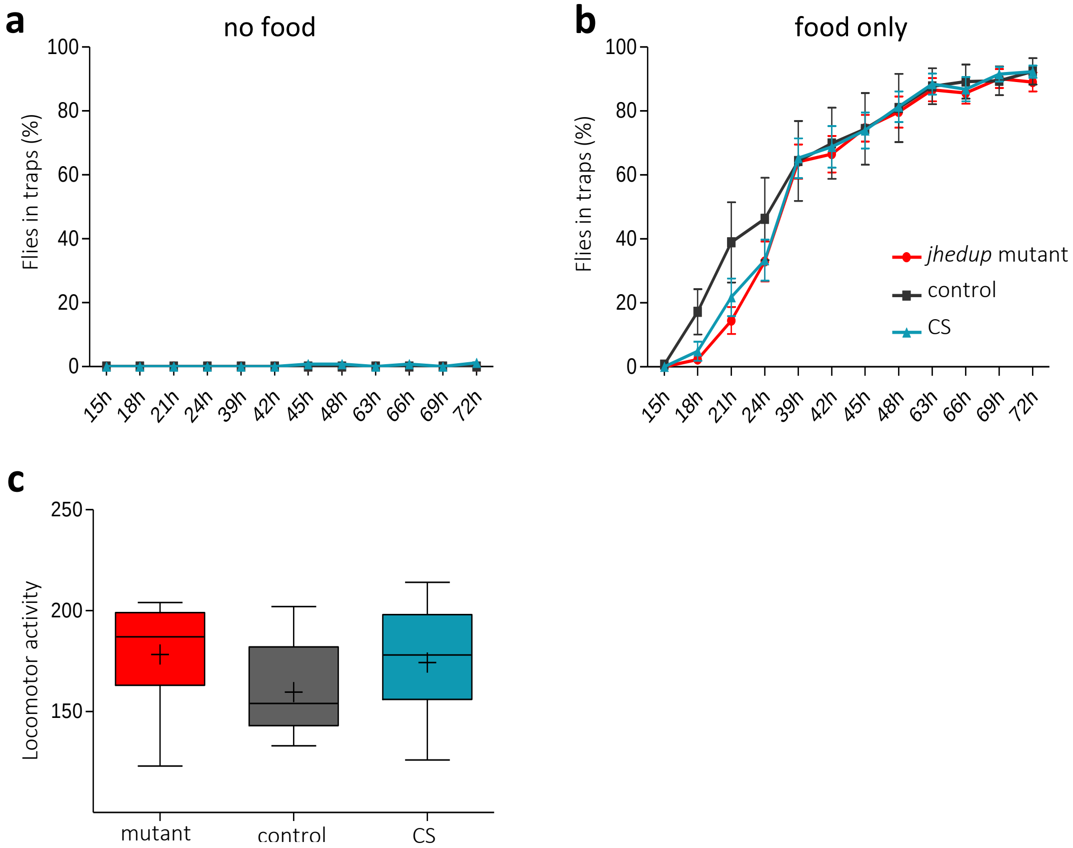

Figure S2. Control tests for impact of *jhedup* mutation on Drosophila's general behavioural response over time and locomotor activity. Behavioural response of non-starved *jhedup* mutant, control and CS females to traps with food (a) and without food (b) over time. Behavioural response is shown as number of flies in traps in percent, mean ± SE (N ≥ 16 traps for each data point ≈240 flies); 2wayANOVA, post-hoc: Bonferroni. (c) Locomotor activity of *jhedup* mutant, control and CS females. Mean ± SE (N = 15 for each genotype); mean indicated as +; 1wayANOVA, post-hoc: Bonferroni's multiple comparison.

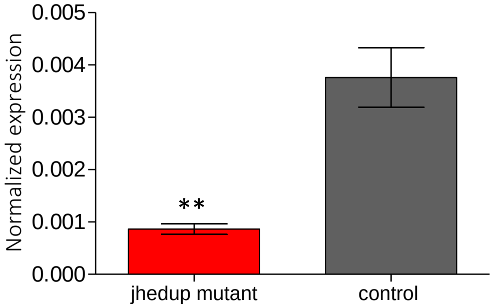

Figure S3. *Jhedup* expression in *jhedup* mutant. Normalized expression of *jhedup* male and female heads of *jhedup* mutant and control line using qPCR (reference gene for normalization of expression: *pgk*). The normalized expression level is indicated as mean ± SE of triplicate biological samples, unpaired t-test, **P ≤ 0.01.

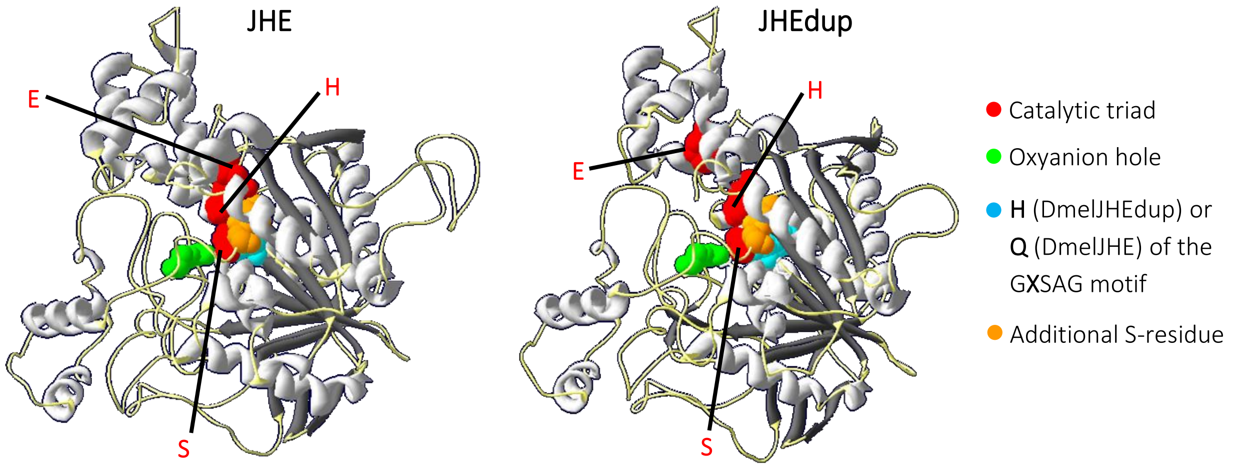

Figure S4. Structural model of DmelJHE and DmelJHEdup. Protein structures were predicted using Phyre2 web portal. The primary structure of DmelJHEdup consists of 559 amino acids while DmelJHE is slightly longer with 579 amino acids. Sheets are colored in dark grey and helices in light grey. Protein structure of DmelJHEdup and DmelJHE is highly similar like orientation of secondary structure elements (helices, sheets), position of Oxyanion hole (green balls) or additional S-residue (pink balls) and size of binding pocket. Differences occur in the position of the glutamic acid residue (E) which is a member of the catalytic triad. Residues of the catalytic triad are indicated as red balls, E: Glutamic acid; H: Histidin; S: Serine.
